# A single-nucleus RNA-sequencing pipeline to decipher the molecular anatomy and pathophysiology of human kidneys

**DOI:** 10.1038/s41467-019-10861-2

**Published:** 2019-06-27

**Authors:** Blue B. Lake, Song Chen, Masato Hoshi, Nongluk Plongthongkum, Diane Salamon, Amanda Knoten, Anitha Vijayan, Ramakrishna Venkatesh, Eric H. Kim, Derek Gao, Joseph Gaut, Kun Zhang, Sanjay Jain

**Affiliations:** 1 Department of Bioengineering, University of California, San Diego, La Jolla, 92093 CA USA; 2 Department of Medicine (Renal), Pathology and Immunology, Washington University School of Medicine, St. Louis, 63110 MO USA; 3 Biological Engineering Program, Faculty of Engineering, King Mongkut’s University of Technology, Thonburi, 10140 Bangkok Thailand; 4 Kidney Translational Research Center (KTRC), Washington University School of Medicine, St. Louis, 63110 MO USA; 5 Department of Surgery (Urology), Washington University School of Medicine, St. Louis, 63110 MO USA

**Keywords:** RNA sequencing, Kidney

## Abstract

Defining cellular and molecular identities within the kidney is necessary to understand its organization and function in health and disease. Here we demonstrate a reproducible method with minimal artifacts for single-nucleus Droplet-based RNA sequencing (snDrop-Seq) that we use to resolve thirty distinct cell populations in human adult kidney. We define molecular transition states along more than ten nephron segments spanning two major kidney regions. We further delineate cell type-specific expression of genes associated with chronic kidney disease, diabetes and hypertension, providing insight into possible targeted therapies. This includes expression of a hypertension-associated mechano-sensory ion channel in mesangial cells, and identification of proximal tubule cell populations defined by pathogenic expression signatures. Our fully optimized, quality-controlled transcriptomic profiling pipeline constitutes a tool for the generation of healthy and diseased molecular atlases applicable to clinical samples.

## Abstract (web summary)

Single-cell studies in solid tissues remain challenging and have benefited from the development of single-nuclei RNA sequencing strategies. Here Lake et al. apply single-nucleus RNA sequencing to human kidney tissues to provide a comprehensive molecular and cellular atlas of the human kidney, with potential implications for the understanding of kidney physiology and disease.

## Introduction

The kidney and lower urinary tract in mammals have undergone unique adaptations during evolution to maintain the composition of blood and homeostasis (water conservation, acid, base and electrolyte balance, red blood cell production, blood pressure regulation). To achieve this, the kidney has a complex cellular architecture consisting of about a million nephrons in humans^1^ that are comprised of more than 40 different cell types^2^. This includes an extensive tubular component that shows a continuous epithelial lining from the beginning of the filtration in the glomerulus, through different segments of the nephron, into the collecting ducts that will ultimately drain into the ureter and bladder. These epithelia provide complex, specialized region-specific roles during this process and interact with a highly complex and heterogeneous niche consisting of: interstitium; a varied vascular network; lymphatics; resident immune cells; the extracellular matrix; and nerves. Defects in this process can have serious health issues, including chronic kidney disease (CKD) and end-stage renal disease (ESRD) that affects >25 million Americans, incurs more than $50 billion in healthcare costs and are significant causes of morbidity and mortality^3–6^. Therefore, fully understanding the cellular composition and associated functional attributes that underlie normal and aberrant kidney function is of high interest for efforts in rebuilding an adult human kidney and for personalized medicine. However, a major impediment to single-cell interrogation of such solid tissues, characterized by high epithelial content and extracellular matrix, remains to be the activation of gene expression artifacts, poor viability and disproportionate cell-type recovery following their enzymatic dissociation. To overcome this, tissues can be effectively reduced to single-nucleus isolates that can provide highly accurate cell-type expression profiles^7–14^ while reducing dissociative artifacts^12^. Here we develop a robust tissue processing and single-nucleus transcriptomic profiling pipeline to interrogate cell-type diversity and establish a molecular “blueprint” of adult human kidney tissue. Our approach demonstrates feasibility of working with limited tissue on a scale compatible with clinical biopsies, with the advantage of concurrent histological registration of tissue content and pathology. Using this approach, our analysis portrays remarkable cellular and molecular heterogeneity and insights into kidney organization, function and disease. Additionally, we identify the expression of ~160 disease-associated genes in discrete kidney cell types, providing new disease targets and unexplored avenues for adaptation or maladaptation to adverse physiology, such as hypertension.

## Results

### A kidney tissue processing pipeline for cell type profiling

**Fig. 1:**
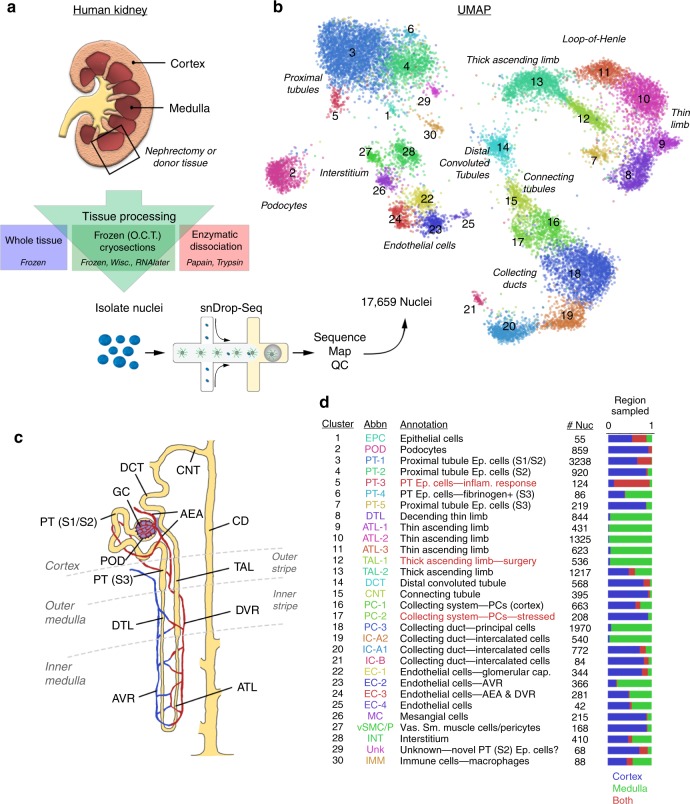
Single-nucleus RNA-seq interrogation of the human kidney. **a** Overview of kidney tissue processing methods tested for nuclei isolation and snDrop-seq. **b** UMAP visualization of 17,659 single nuclei (15 individuals, Supplementary Data 4) passing QC filtering (Supplementary Fig. 2a). Source data are provided as a Source Data file. **c** Schematic of a juxtamedullary nephron^36^ showing relevant cell types and associated vasculature. **d** Table of single-nucleus clusters shown in (**b**), indicating associated cell-type annotations, number of nuclei per cluster and relative origination from samples encompassing the cortex, medulla or both (Supplementary Data 6 and provided as a Source Data File). UMAP uniform manifold approximation and projection

Comprehensive cell-type discovery in adult human kidney requires a robust approach that can allow accurate reflection of physiological function while minimizing confounding influences from tissue manipulation. To develop a method that can be highly reproducible, applicable to biopsy-scale tissue, and maximizes assay success rates for adult kidney, we established a tissue processing pipeline for assessing single nuclei using snDrop-seq^8^ (Fig. 1a). To this end, we procured kidney tissue from tumor-free regions of nephrectomies and discarded deceased donor kidneys having detailed preanalytical parameters (Supplementary Data 1) and compared multiple preservation and processing methods (Fig. 1a, Supplementary Data 2). This included cryopreserved and fresh tissues that were partially dissociated enzymatically using methods employed for single whole-cell studies (papain/collagenase denoted dissocPC; trypsin/collagenase denoted dissocTC). Interestingly, we found that only nuclei from cryosectioned (~40 µm thick) optimal cutting temperature (O.C.T.)-embedded frozen tissues and dissociated fresh tissues were compatible with snDrop-seq. This indicated a possible susceptibility of the kidney nuclear membranes to damage during processing, including stress forces needed for isolation from larger kidney tissue segments. Consistent with this, we found that fluorescent cytometry, successfully employed in the purification of brain nuclei^7,8^, impeded snDrop-seq success on kidney nuclei (Supplementary Fig. 1a). Therefore, both fresh dissociated and frozen cryosectioned kidney tissues retained sufficient nuclear RNA levels for snDrop-seq assays. We achieved further improvement in cryosectioned tissue quality through preservation in RNAlater (CryoR), compared with Wisconsin Cold Storage Solution (CryoW) or maintenance on dry ice or at −80 °C (CryoF) until nuclei were isolated (Supplementary Fig. 1b). This permitted greater flexibility in sample processing, both in terms of the physical locations at which different steps can be performed and in the timing for execution of the molecular assay. The ability to use cryosectioned material in these assays further permits histological verification and spatial registration on adjacent sections to evaluate the composition of tissue being used for downstream analysis (Supplementary Data 1). Therefore, these results have important implications for highly scaled and collaborative tissue interrogation efforts across multiple sample procurement and tissue interrogation sites.

**Fig. 2:**
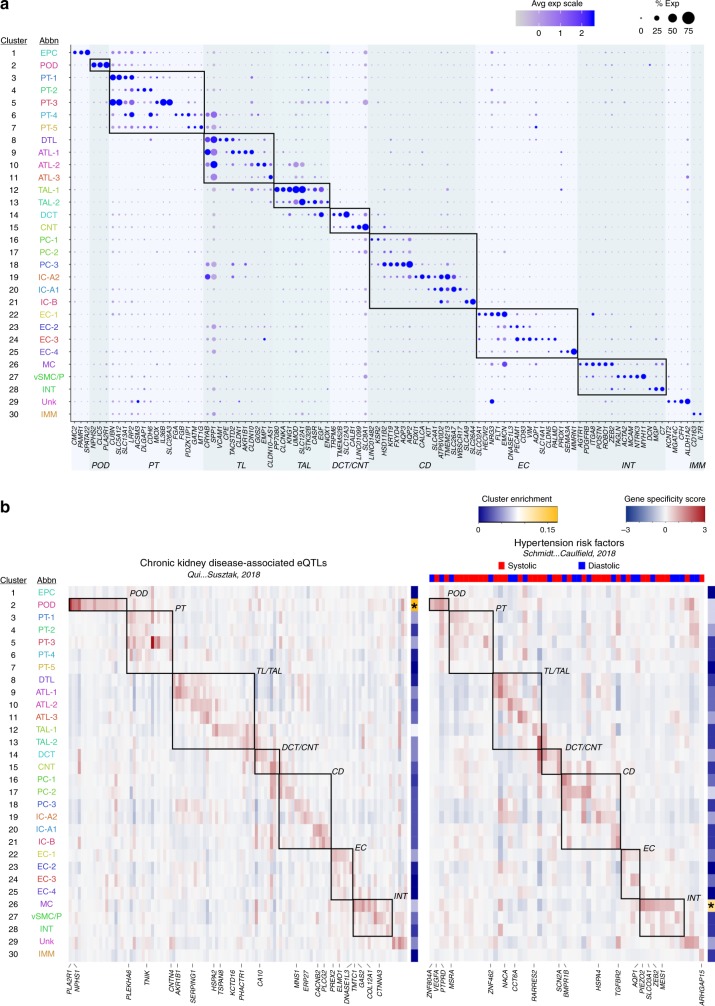
snDrop-seq clusters show distinct expression profiles. **a** Dot plot of select average gene expression values (log scale) and percentage of nuclei expressing these genes within each cluster (Fig. 1b) for known (bold) and newly discovered cell-type marker genes (see Methods, Supplementary Data 7). **b** Heatmap showing cluster-specific gene specificity scores (see Methods) for differentially expressed genes associated with CKD eQTL^21^ and hypertension risk loci^22^ (systolic and diastolic blood pressure variants are indicated). The genes corresponding to these clusters are in Supplementary Data 8. Asterisks indicate higher cluster enrichment (proportion of differentially expressed genes associated with each cluster) of CKD-associated eQTLs in POD and hypertension risk factors in MC. Source data are provided as a Source Data file. CKD chronic kidney disease, eQTL expression quantitative trait loci, POD podocytes

To determine the performance of single-nucleus RNA-seq expression estimates in generating an adult human kidney cell-type molecular atlas, data from 27 different experiments and 15 different individuals from two different institutions (Washington University and University of Michigan through KPMP consortium) were combined for analysis (Supplementary Data 2−3). After quality control filtering (Supplementary Fig. 2a), 17,659 nuclei sequenced to a depth for 1082 unique transcripts on average detected per nucleus were found to resolve into 30 distinct cell clusters using PAGODA2^8,15^ (Fig. 1b, Supplementary Data 4). These represented common and rare cell types found in the kidney, with the expected spatial distribution across cortex and medullary zones, as well as known and newly discovered kidney marker gene expression profiles (Figs. 1c, 2a, Supplementary Fig. 2b–c, Supplementary Data 5−7). We further confirmed stability of these cluster assignments over varying clustering parameters, consistent with the distinction of biologically relevant populations (Supplementary Fig. 3). In this way, we identified all major components of the glomerulus epithelium, endothelium and interstitium. This includes: podocytes (POD); uncharacterized epithelial cells (EPC); glomerular capillaries (EC-1, GC) and mesangial cells (MC) (Fig. 1c). We also detected several renal tubule populations extending from the proximal tubules (PT-1-5) to the loop of Henle (LOH), including the descending thin limb (DTL), thin ascending limb (ATL1-3) and thick ascending limb (TAL-1-2), and finally through to the distal convoluted tubule (DCT) (Fig. 1c). The DCT connects to the collecting duct (CD) through a transitory population of connecting tubule cells (CNT) that showed graded expression of markers for the DCT and CD (Fig. 2). Our data delineate the complexity of the collecting system cell types, including several principal cells (PC-1-3) and intercalated cells (IC-A1-2, IC-B) consistent with prior studies^16,17^, as well as molecularly distinct populations residing between the cortex and medulla not previously recognized. In addition to the major proximal and distal nephron populations, we found: endothelial cells associated with afferent/efferent arterioles and peritubular capillaries (AEA, EC-3) and both the ascending and descending vasa recta (AVR, DVR; EC-2-3); mesangial cells (MC, cluster 26), vascular smooth muscle cells or pericytes (vSMC/P, cluster 27), interstitial cells (INT, cluster 28); and tissue-resident macrophages (IMM, cluster 30). Correlation analysis revealed expected cell-type associations (Supplementary Fig. 4a, b) and a high correspondence with major cell types reported previously in mouse and human studies (Supplementary Fig. 4c)^17,18^. However, our snDrop-seq method permitted a greater resolution of nephric cell types and subtypes compared to even large-scale human studies^19^, providing cluster resolution more accurately representative of expected histological composition.

Different regions and cell types of the kidney play unique and key roles in regulating blood pressure by maintaining volume, electrolyte, and acid-base homeostasis. Dysfunctions can be a significant cause of CKD and hypertension. As such, several renal sites are targets for existing antihypertensive treatments, including the LOH (loop diuretics), DCT (thiazide diuretics), and the CD (potassium sparing diuretics). In addition, inhibitors of the renin angiotensin aldosterone system (RAAS), that regulates blood pressure and fluid balance, show cardiovascular and renal benefits, partly through targeting proteins expressed in specific renal tubule segments^20^. However, despite advances, hypertension remains extremely common and uncontrolled blood pressure is still associated with devastating cardiovascular and renal outcomes. We next leveraged emerging large-scale genome-wide data identifying target loci for these disorders to interrogate their cell-type-specific expression. Our clusters showed significant (adjusted *p* value < 0.05, Wilcoxon rank sum test) differential expression of 111 out of 674 genes identified as expression quantitative trait loci (eQTL) associated with CKD^21^ and 56 out of 220 genes that were recently linked to hypertension risk from genome-wide analysis of over a million individuals^22^ (Fig. 2b, Supplementary Data 8). Interestingly, we observed restricted expression for several disease-associated genes in specific cell types, with PODs and MCs showing the highest enrichment for CKD and hypertension risk loci, respectively (Fig. 2b, Supplementary Fig. 4d). This observed cell-type specificity, also shown for mouse single-cell data^21^, suggests multiple unique functionalities may contribute to dysfunctional kidney physiology and hypertension. While validation would be needed to confirm any causal roles, this analysis can aid in discovery of potentially physiologically important factors. For instance, we find CNT-specific expression of the gene encoding the voltage-gated sodium channel SCN2A, previously found in the brain and critical for post-natal survival^23^, yet having no known function in kidney. Perhaps SCN2A has an uncharacterized role in CNT function, an area important in salt regulation. Therefore, our single-nucleus interrogation of the kidney finds not only coverage of major cell types, but also subpopulations indicative of finer resolution compared to prior studies on the adult human kidney^18,19^, and cell types with enriched expression of CKD and hypertension-associated loci.

To better understand subpopulations identified in our data and assess biological from technical variations, we examined more closely several metadata, quality assessment and quality control metrics (Supplementary Figs. 5−6). Mitochondrial transcripts (MT), while not expressed in nuclei and excluded from downstream analyses, were found in variable quantities (calculated prior to MT transcript removal) associated with single-nucleus data (Supplementary Fig. 5a–c), indicative of mitochondrial association with the nuclear membranes. Given that elevated MT can be associated with lower cell viability^24^, it was expected that the level of MT in nuclei data may also reflect the quality of the tissues during processing (Supplementary Fig. 5a). Consistently, we observed the fewest MTs and stress-induced artifacts in cryoR samples compared to cryosections processed differently (cryoW, cryoF) or fresh dissociated samples (dissocPC, dissocTC), while global gene and transcript levels appeared unaffected (Supplementary Figs. 5a, 6). Interestingly, both the TAL population (TAL-1, cluster 12) and the S3 PT (PT-5, cluster 7) (Supplementary Fig. 5b, c), regions with high metabolic demand and prone to ischemia^25^, showed MT enrichment consistent with the nephrectomy procedure-related warm ischemia (Supplementary Fig. 5c, d). Thus, knowledge of preanalytical tissue procurement and processing parameters enabled better interpretation and analyses of the snRNA-seq data. This allowed us to recognize artifacts and determine cryoR preservation of archived O.C.T. frozen sections as the optimal method for snRNA-seq interrogation.

We additionally examined clusters for potential sources of variations (region, batch, individual, collection, sex) in our data (Supplementary Fig. 5d, e). While medullary clusters, associated with the LOH and CDs, were covered mostly from samples from three individuals, cortical clusters were covered by 14 individuals with negligible batch effects. However, we did find a PT cluster that was predominantly derived from a single individual (PT-3, cluster 5). This cluster was enriched in inflammatory genes (Supplementary Fig. 6) and could reflect an altered state of this PT population by underlying disease or procedure. Overall, averaged expression values for cortex data was highly consistent between experiments, with slightly greater correlation values within individuals compared to between different individuals (Supplementary Fig. 6a, b). Technical reproducibility within snDrop-seq experiments was found to be very high, as were expression values between the different sexes (Supplementary Fig. 6b) and between samples obtained from different tissue procurement sources (Supplementary Fig. 6c). To examine the effect of the different tissue processing methods used, we calculated average expression values for each condition for all data, cortex and medulla data separately, and within specific cell clusters or individuals (Supplementary Fig. 6e–j). In each case, there was a high correspondence, with cryoW showing the lowest correlation in gene expression values. Differential expression analysis between the different methods revealed a stress response signature (including *FOS, FOSB, DNAJB1*) that was specifically associated with whole cell dissociative methods and which could be found in most of the cell types detected (Supplementary Fig. 6k). However, one CD cluster (PC-2, cluster 17) that showed stress response gene expression (Supplementary Fig. 6k) was found to be mostly attributed by dissocP/T processing methods (Supplementary Data 6, Supplementary Fig. 5d, e) and as such likely represented a dissociation artifact. CryoR samples showed the fewest expression artifacts while also exhibiting higher experimental success rates (>90%) (Supplementary Data 2), thereby providing an optimized tissue processing through to snDrop-seq pipeline applicable to large-scale single-cell interrogation studies on solid tissues (see Methods).

### Characterization of known and new kidney cell types in PTs

**Fig. 3:**
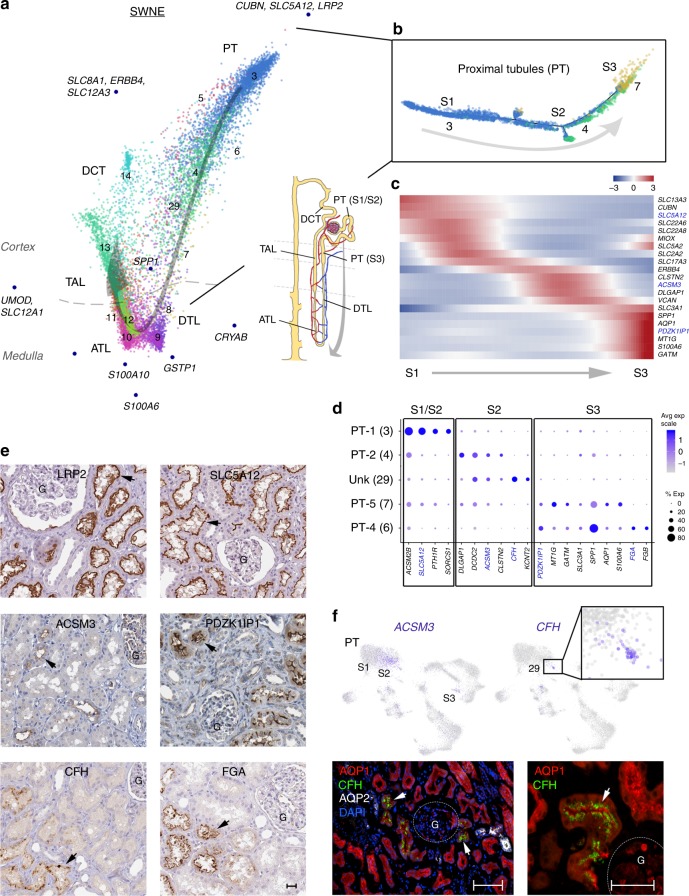
Proximal tubule cell populations. **a** SWNE analysis of tubule clusters (3–14, 29) for cell types indicated (PT, DTL, ATL, TAL to DCT). Arrow indicates progression of lineages corresponding with expected spatial ordering in vivo. SWNE factor-associated genes (Supplementary Data 9) and relative medulla/cortex originations are indicated. **b**. Trajectory analysis of main PT clusters supporting their S1, S2 and S3 PT segment identities. **c** Heatmap of expression (row Z-scores) for genes differentially expressed along the proximal tubule trajectory (Supplementary Data 10) shown in (**b**). **d** Dot plot showing select segment-specific marker gene expression (averaged log scale values and percentage of expressed nuclei), localizing the PT clusters Unk and PT-4 to S2 and S3 segments. **e** Protein immunostaining (Human Protein Atlas^49^, Supplementary Data 16) for select PT markers, including general PT (LRP2), segment-specific (highlighted in **c**), and Unk/PT-4 cluster (CFH and FGA) markers. Arrows indicate representative protein localization. Scale bar indicates 25 µm. **f** UMAP plots as in Fig. 1b showing expression level (scaled from low—gray to high—blue) of S2 segment marker *ACSM3* and Unk cluster 29 marker *CFH*. Lower panels show protein fluorescent immunostaining (Supplementary Data 17) for CFH in a subset of PT co-stained with AQP1 expression in PT, but not the PC marker AQP2 (arrows). Location of glomeruli (G) and scale bars (left panel—100 µm; right panel—50 µm) are indicated. Source data for (**a**−**d**, **f**) (UMAP plots) are provided as a Source Data file. UMAP uniform manifold approximation and projection, SWNE Similarity Weighted Nonnegative Embedding

After discovery of clusters with possible tissue processing, collection or patient-derived artifacts (clusters 5, 12, 17), we analyzed the remaining populations to glean insights into molecular anatomy and biology of the adult human kidney. During fetal development, pretubular aggregates proliferate and mature into anatomically distinct tubular segments of the nephron, including PT, LOH and DCT. How these segments are molecularly interconnected in the mature kidney remains less well characterized. Similarity Weighted Nonnegative Embedding (SWNE) analysis^26^ of renal tubular data revealed a spatial organization of tubular component nuclei progressing from PT, through the thin limb (TL) and TAL to the DCT, indicating a continuum of gene expression progressing between the different nephron segments (Fig. 3a, Supplementary Figs. 7−8, Supplementary Data 9). The three main PT clusters (PT-1, PT-2, PT-5) further showed ordering consistent with known S1, S2 and S3 PT segments, that was supported by an unbiased trajectory analysis^27^ of the associated nuclei (Fig. 3b). Differential expression along this trajectory revealed potential segment-specific expression signatures associated with differential uptake and metabolic processes (Fig. 3c, Supplementary Fig. 8b, Supplementary Data 10), and permitted identification of distinct markers, including *PDZK1IP1* that showed consistent protein localization to specific subsets of the PTs consistent with the S2 segment marker *ACSM3*^28^ (Fig. 3d–f). However, genes encoding anion and cation transporters expected to be enriched in the S2 segment (including *SLC22A6* and *SLC22A8*, Supplementary Data 5) were found within the PT-1 (3) population, indicating: (1) PT-1 may represent a mixture of S1 and early S2 cells; (2) a possibility for underlying differences in the human proximal tubule segments compared with lower mammals; (3) some discrepancy in cell types defined molecularly compared to those described from physiology. In addition to these main PT clusters, we also found two additional S2 and S3 PT populations, Unk (29) and PT-4 (6) (Fig. 3d). Unk (29), while not showing obvious PT marker expression (e.g. *LRP2* or *CUBN*, Fig. 2a), did show expression consistent with S2 segment markers genes (Fig. 3d). This population also showed distinct expression of the *complement factor H* (*CFH*) gene, encoding for a component of the innate immune system involved in the defense against invasive pathogens^29^ and that was found localized to a subset of the proximal tubules similar to that seen for ACSM3 (Fig. 3e, f). Interestingly, *CFH* genetic mutations have been associated with kidney pathologies, including atypical hemolytic uremic syndrome (aHUS), C3-glomerulopathy (C3G) and end-stage kidney disease (ESKD)^30–32^. These findings may implicate a subset of S2 PTs in the defense or clearance of affected tubular cells for maintenance of normal kidney function. In addition to this CFH^+^ population, we also find PT-4 (6) showing distinct expression of *LRP2* (Fig. 2a), having an expression signature associated with the S3 segment, and that uniquely expressed *Fibrinogen Alpha* (*FGA*) and *Beta* (*FGB*) *Chain* genes (Fig. 3d). Consistently we find FGA protein localized to a subset of the proximal tubules (Fig. 3e). *FGA/FGB* genes encode for fibrinogens that may play an important role in inflammation and regeneration following kidney injury^33^. However, if in excess, these may impede recovery, with elevated urinary fibrinogen levels associated with progression of CKD to ESKD^34^. Considering the S3 segment has been found to be prone to acute kidney injury, *FGA/FGB* may also contribute to AKI progression to CKD. Therefore, we identified and validated two PT populations with defining gene expression profiles that may represent possible important roles in acute and CKD.

### Defining proximal nephron to the collecting system transition

**Fig. 4:**
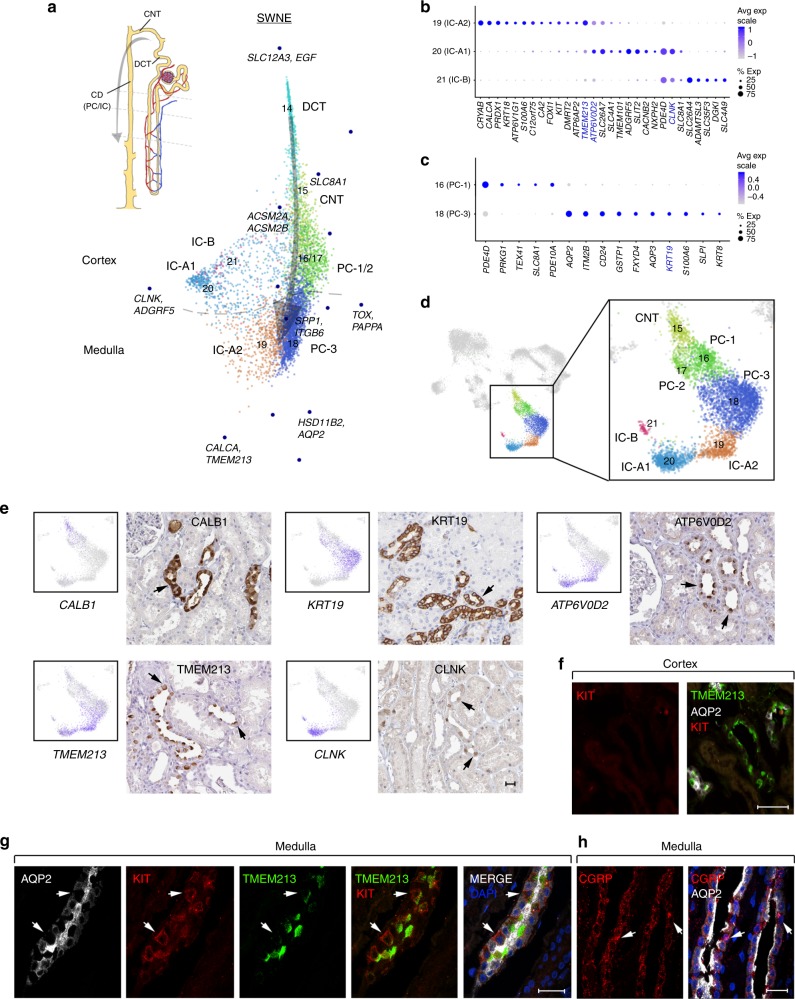
Cellular and molecular diversity of the collecting ducts. **a** SWNE analysis of cell clusters (14–21) from distal nephric tubule progressing through collecting duct (DCT, CNT, PC, IC) showing spatial distributions comparable to that expected in vivo. SWNE factor-associated genes (Supplementary Data 9) and relative medulla/cortex originations are indicated. **b** Dot plot showing select (see Methods) IC marker gene expression (averaged log scale values and percentage of expressed nuclei). **c** Dot plot showing select (see Methods) cortex and medullary collecting system PC marker gene expression (averaged log scale values and percentage of expressed nuclei). **d** UMAP as in Fig. 1b showing clusters used in (**a**). **e** Protein immunostaining (Human Protein Atlas^49^, Supplementary Data 16) for the select PC and IC markers highlighted in (**b**) and (**c**) showing protein localization within the CNT/CD (arrows). UMAP plots show corresponding RNA expression levels (scaled from low—gray to high—blue). Scale bar indicates 25 µm. **f** Protein fluorescent immunostaining (Supplementary Data 17) for KIT, IC marker TMEM213 and PC marker AQP2 in the cortical collecting ducts. Scale bar indicates 50 µm. **g** Protein fluorescent immunostaining for KIT, IC marker TMEM213 and PC marker AQP2 in the medullary collecting ducts. Arrows indicate representative AQP2-negative cells showing colocalization of KIT and TMEM213. Scale bar indicates 25 µm. **h** Protein fluorescent immunostaining showing CGRP enrichment in AQP2− medullary collecting duct cells (arrows). Scale bar indicates 20 µm. Source data for (**a**−**d**) are provided as a Source Data file. UMAP uniform manifold approximation and projection, SWNE Similarity Weighted Nonnegative Embedding

The ureteric bud, a derivative of the anterior intermediate mesoderm, produces the collecting ducts (PC, IC-A and IC-B cells) and cap mesenchyme, while a derivative of the posterior intermediate mesoderm produces the nephron segments (POD, PT, LOH, DCT, CNT)^35^. Examination of LOH populations revealed four distinct TL clusters, with markers consistent with potential DTL (*AQP1*^*+*^*, ID1*^*+*^), and with ATL-1-3 (*AQP1*^*−*^*CLDN10*^*+*^) showing expression consistent with the pre-bend (epithelium similar to ascending limb), post-bend and ATL segments, respectively^36,37^ (Supplementary Data 5, Supplementary Fig. 8c). This is further supported by spatial distribution of these populations using SWNE (Fig. 3a). The subsequent joining of DCT with CDs through CNT is critical for proper functioning of the kidney and maintaining a contiguous urinary tract. However, molecular signals that define these transitions in the adult kidney remain poorly resolved. Analysis of DCT, CNT, PC, IC clusters using SWNE revealed that transitions from DCT to CNT through to CDs in the cortex was predominantly represented by IC-B, IC-A1 and PC-1, and further progression into the medulla was represented mainly by PC-3 and IC-A2 (Fig. 4a). Closer examination of known ion channels critical to the function of the DCT/CNT (Supplementary Fig. 9a, b) showed consistent progressive expression in these subpopulations. However, our data were unable to molecularly distinguish separate DCT subpopulations (DCT1 and DCT2) found previously^38^. These might underlie *SLC12A3*^*+*^*/SLC8A1*^*−*^*/HSD11B2*^*−*^ *or SLC12A3*^*+*^*/SLC8A1*^*+*^*/HSD11B2*^*+*^ cells found transitioning between these populations in DCT cluster 14 (Supplementary Fig. 9c). CD subpopulations mainly showed gene expression profiles (Fig. 4b–e, Supplementary Fig. 9d, Supplementary Data 11−12) consistent with CD physiology, as seen by expression of genes encoding the chloride/bicarbonate exchanger Pendrin (SLC26A4) in IC-B cells^39^ and water channels AQP2 and AQP3 in the PC-3 cells^40^. SWNE revealed the genes that most heavily contributed to the DCT to CD progression, with *SLC8A1*, *AQP2/HSD11B2*, *CALCA/TMEM213* and *CLNK/ADGRF5* driving to CNT/PC-1, PC-3, IC-A2 and IC-B/IC-A1 populations, respectively (Fig. 4a, Supplementary Fig. 7). Among the two PC cell types, PC-1 was mainly found in the cortex and its gene expression highly correlated with the medullary CD PC-3 cluster and CD populations from recent studies^17,18^ (Supplementary Fig. 4c) suggesting its assignment as CD. However, the PC-1 cluster also exhibited expression of the CNT marker *SLC8A1* in a subset of cells with little overlap with *AQP2*, unlike observations in rodent CNT where these marker genes strongly overlap^28^. This suggests possible divergence in this population in humans or that these cortical PCs might populate late CNTs and cortical CDs, resolution of which will require further sampling. Expression of the typical CD PC marker *AQP2* was also seen in a subset of IC-A2 cells, confirming the presence of IC-PC hybrid cells that have been observed in mice^16^. Furthermore, the above discussed cluster 17 (PC-2), showing stress response gene activation, appeared to arise from PC-1, indicating this population of cortical collecting system cells might be particularly susceptible to enzymatic dissociation-associated artifacts.

While IC subpopulations showed distinct expression profiles (Fig. 4b), IC-A1 shared markers with both IC-A2 and IC-B, supporting cell-type and functional transitions in IC populations from cortical to medullary CDs. However, the IC-A2 cluster did show unique expression of two biologically relevant genes, *KIT* and *CALCA. KIT* encodes a receptor tyrosine kinase that promotes self-renewal and survival in a number of tissues. Recent evidence showed Kit as a surface receptor expressed on mouse IC-A cells^16^ and that Kit^+^ cells isolated from the kidney were capable of promoting repair and attenuation of POD injury in mice^41^. Using immunofluorescence, we confirmed KIT protein expression specifically within ICs of the medulla that coexpressed TMEM213, but not the PC marker AQP2 (Fig. 4f, g). This population also showed specific expression of *CALCA* that encodes for calcitonin gene-related peptide (CGRP) that is well-known for its roles in mediating responses to noxious stimuli, pain and inflammation in the sensory nervous system^42^. CGRP is mainly expressed by sensory neurons (and some immune and epithelial cells) and acts as a potent vasodilator if released at nerve terminals innervating the blood vessels. However, a potential role for CGRP has not yet been reported in the kidney. Using immunofluorescence staining, we confirmed CGRP expression in CDs, although detected in both intercalated and principal cells (Fig. 4h). Thus, expression of KIT and CGRP in IC-A2 cells may poise them for several intriguing roles needing further investigation: sensing or mediating inflammatory or sensory responses to uropathogenic infections in the CD; modulation of the medullary blood flow by acting on the medullary blood vessels; and/or promoting renewal or survival following injury.

### Defining endothelial and interstitial cell populations

**Fig. 5:**
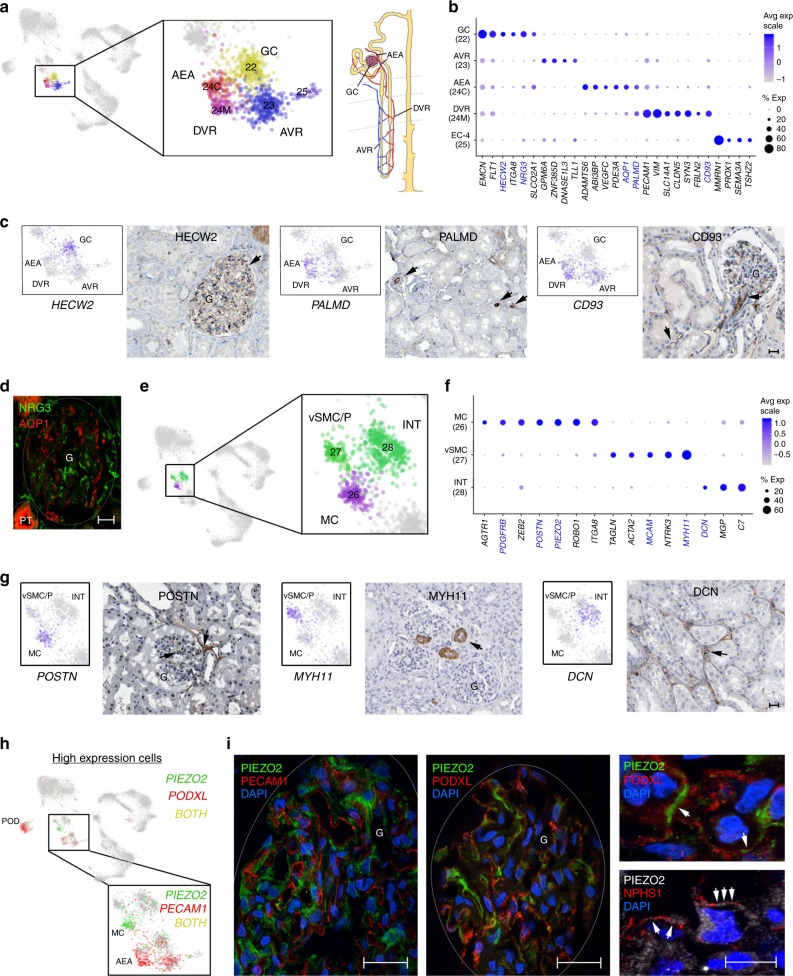
Resolution of endothelial and interstitial lineages. **a** UMAP as in Fig. 1b showing EC clusters (22–25) and a schematic of the nephron vasculature. **b** Dot plot showing select (see Methods) EC marker gene expression (log scale values and percentage of expressed nuclei). **c** Protein immunostaining (Human Protein Atlas^49^, Supplementary Data 16) for the select EC markers highlighted in (**b**). Arrows indicate representative protein staining. UMAP plots show corresponding RNA expression levels (scaled from low—gray to high—blue). Scale bar indicates 25 µm. **d** Protein fluorescent immunostaining (Supplementary Data 17) for GC-specific NRG3 and AQP1 expressed in AEA (highlighted in **b**) and PT. Scale bar indicates 25 µm. **e** UMAP as in Fig. 1b showing interstitial clusters (26–28). **f** Dot plot showing select interstitial marker gene expression (see Methods) and within-cluster detection rates. **g** Protein immunostaining (Human Protein Atlas^49^, Supplementary Data 16) for the select markers highlighted in (**f**). Arrows indicate representative protein staining. UMAP plots show corresponding RNA expression levels (scaled from low—gray to high—blue). Scale bar indicates 25 µm. **h** UMAP plots as in Fig. 1b indicating high expressing cells for PIEZO2 (green) and PODXL or PECAM1 (CD31) (red), or both PIEZO2 and PODXL or PECAM1 together (yellow). **i** Protein fluorescent immunostaining (Supplementary Data 17) for MC-specific PIEZO2, vascular marker PECAM1 and POD markers PODXL and NPHS1. Arrows indicate representative PIEZO2 protein localized to the MC membrane adjacent to POD cells (PODXL) and localized between POD processes (NPHS1). Location of glomeruli (G) and scale bars (left and middle panels—25 µm, right panel (top and bottom)—10 µm) are indicated. Source data for (**a**, **b**, **e**, **f**, **h**) are provided as a Source Data file. UMAP uniform manifold approximation and projection

The interplay between endothelial (Fig. 5a–c, Supplementary Fig. 10) and interstitial populations (Fig. 5d–g, Supplementary Fig. 11a) is critical for renal functionality. This is especially evident in the renal corpuscle where glomerular capillaries and mesangial cells, specialized glomerular pericytes, maintain appropriate filtration capacity for fluids moving into the proximal portion of the nephron. Consistently, we identified an *EMCN*^*+*^*/HECW2*^*+*^*/CD93*^*−*^ GC cell population (EC-1, Cluster 22) juxtaposed spatially with *POSTN*^*+*^/*PDGFRB*^*+*^*/ITG8A*^*+*^*/PIEZO2*^*+*^ MCs (Cluster 26) within the glomeruli, as indicated by immunostaining of their associated proteins (Fig. 5c, g, i). We identified *PIEZO2*, encoding a stretch-gated ion channel involved in mechano-sensation^43^, as a new marker expressed in MCs (Fig. 5f). High-resolution confocal immunofluorescence microscopy with POD and EC markers support PIEZO2 protein expression in MCs adjacent to the podocytes and endothelial cells (Fig. 5h–i, Supplementary Fig. 11b). PIEZO2 expression in MCs suggests its possible role in mediating responses to mechanical distensions during variations in blood flow or pressure. Interestingly, *PIEZO2* has recently been identified as a novel hypertension locus^22^ (Fig. 2b and Supplementary Data 8). Our identification of *PIEZO2* gene expression by mRNA and protein analysis in MCs is of particular importance given that high blood pressure represents one of the leading causes of kidney failure and that MCs play a key role in regulating blood flow and surface area of podocytes for filtration.

In addition to *CD93*^*−*^ GCs, we identified *CD93*^*+*^ vasculature leading into and out of glomeruli (AEA) and progressing into the DVR (EC-3 or cluster 24) and finally to the AVR (EC-2, cluster 23) (Fig. 5a), the latter two marked by expression of *PALMD* and *DNASE1L3*, respectively (Fig. 5b, Supplementary Data 13). Consistently PALMD protein localized to vascular endothelium lined by vSMCs that is characteristic of the DVR, but not the AVR (Fig. 5c). The EC-3 population could further be subdivided based on spatial origination of nuclei from either the cortex or medulla (clusters 24C or 24M), permitting finer resolution of cortical vessels from DVR ECs (Fig. 5a), demonstrating their distinct expression profiles (Fig. 5b, Supplementary Fig. 10b, Supplementary Data 13). This includes enrichment of transcripts for *AQP1* in the blood vessels, which was supported by AQP1 protein localization (Supplementary Fig. 10c). This reinforces an important role for AQP1 in ensuring that water reabsorbed by PT segments and LOH can transport into the blood via cortical capillaries and DVR. In addition to these known vascular cell types, we additionally detected a *MMRN1*^+^/*PROX1*^+^ endothelial population that could represent potential lymphatics or capillaries due to their reported expression in endothelia of other organs^44,45^. However, immunostaining with MMRN1, a secreted protein, labeled rare cortical vessels distinct from D240-labeled lymphatics (Supplementary Fig. 11c) and PROX1 immunostaining was unsuccessful on our tissues due to nonspecific staining. Interestingly, MMRN1-positive immunolabeling was also detected in rare cells of the PTs (Supplementary Fig. 11c). Since no appreciable level of *MMRN1* transcripts were detected in our PT clusters (Fig. 2a), this suggests possible transport of the secreted protein from vessels into juxtaposed PT cells. Characterization and function of these unique *MMRN1*^+^ cells in endothelium, representing less than 0.1% of adult kidney cells in our assay, requires further studies.

In addition to the MC population, we also detected *DCN*^*+*^ interstitial fibroblasts (INT) and *TAGLN*^*+*^*/MYH11*^*+*^*/MCAM*^*+*^ vSMC/P showing distinct expression profiles (Fig. 5e, f, Supplementary Fig. 10b, Supplementary Data 14), as confirmed by their associated protein immunostainings (Fig. 5g, Supplementary Fig. 11a). However, known specialized subpopulations, including the REN^+^ cells that are known to associate with the juxtaglomerular apparatus, were only rarely detected within the vSMC/P cluster based on *REN* transcript levels (Supplementary Fig. 11a). This indicates that deeper sampling would be needed for greater diversification of interstitial subpopulations detected using this method.

### Insights into heterotypic interactions

**Fig. 6:**
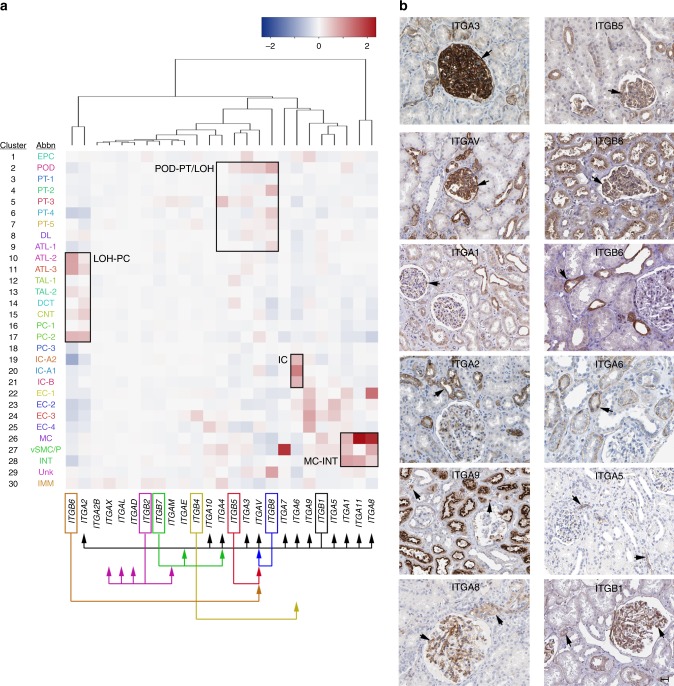
Integrin expression dynamics in the kidney. **a** Heatmap of averaged scaled gene expression values for integrins detected in the adult human kidney clusters. Enrichment for specific integrins and their indicated binding partners (arrows) were found for specific cell types or nephron segments. Source data are provided as a Source Data file. **b** Protein immunostainings (Human Protein Atlas^49^, Supplementary Data 16) for select integrins that showed cluster-enriched RNA expression shown in (**a**). Arrows indicate associated cell types/structures showing enrichment predicted from snDrop-seq data (**a**). Scale bar indicates 25 µm

There are several examples supporting receptor-ligand signaling mechanisms in different cell types of our data. We focused on integrins given their importance as regulators of homeostasis. Integrins are critical receptors that mediate attachment of the cell cytoskeleton to the ECM to promote cellular processes of movement, survival, differentiation and proliferation^46^. These functionalities rely upon the distinct specificities of heterodimeric α and β subunit combinations to ECM components, including: vitronectin, collagen, laminin and fibronectin (via the Arg-Gly-Asp or RGD motif). Imbalances in integrin levels can directly influence matrix deposition and intracellular signaling in disease. For example, α3β1 associations are important for POD integrity and preventing proteinuria, and feedback interactions between ɑ1β1 and ɑ2β1 play important roles in regulating collagen levels in MCs. Given this, and a possible contribution of integrins to fibrosis in acute and chronic kidney diseases, we examined their expression in the distinct cell types and subtypes identified in our data (Fig. 6). From this we found cell-type specificity for major integrins along the different nephron segments and insight into their relevant ECM ligands. PODs showed high expression of genes encoding ITGA3 for laminin binding, ITGA1 for collagen binding, and ITGAV-ITGB5 for fibronectin/vitronectin binding. Alternatively, we found possible ITGAV-ITGB8 (for binding to TGFβ-latency associated peptides) poised for MC matrix signaling. The LOH, DCT, CNT and PCs were enriched in transcripts for ITGA2 (collagen) and ITGB6 (RGD domain binding), while ICs showed high expression of transcripts for ITGA6 (laminin binding), highlighting potential signaling mechanisms in these different cell types in response to the surrounding ECM. Outside the nephron, EC clusters showed highly specific expression of the gene encoding ITGA9 (for binding to non-RGD ligands such as ADAMS, OSN and VEGF) while the mesenchymal and interstitial cells showed high expression of genes for ITGA1 (collagen), ITGA11 (collagen) and ITGA8 (fibronectin/vitronectin). These expression patterns suggest pairing of α1β1 and α11β1 in MCs for their adherence to the collagen matrix in the glomerular basement membrane (GBM) and for interaction with podocytes, while α8β1 may permit transduction of signals from the mesangial matrix. Therefore, these diverse integrin expression profiles provide a better understanding of the interaction of major kidney cell types with their milieu, as well as insights into how these might be disrupted in disease.

## Discussion

We provide an optimized single-nucleus RNA sequencing pipeline for clinical specimens with extensive quality assessments from tissue procurement through to single-cell transcriptomic analyses (Supplementary Fig. 12). This tissue processing and interrogation work-flow is applicable to even limiting amounts of challenging solid tissue samples, thereby maximizing interpretable data while minimizing stress artifacts. Application on adult human kidney has permitted extensive cell-type discovery, including a spatial resolution of nephric subsegments and supportive cell populations that has not yet been observed. This includes the molecular events underlying tubular transition states and insights into anatomical and physiological organization. Further, we find PT populations mapping to specific segments that have demonstrated expression of possible renal pathogenesis-associated genes (*CFH, FGA*), demonstrating the ability of our assay to identify molecular links for distinct cell types that enhances our understanding of renal function and disease. This is further supported by the discovery of a stretch-gated ion channel *PIEZO2*, specifically expressed in glomerular MCs, that maps to a validated hypertension locus^22^, providing a possible link between responses to mechanical changes due to variations in blood pressure, glomerular filtration and kidney failure. The diversity of integrin expression provides further insights into the potential ligand interactions for specific kidney cells that may serve as a benchmark for understanding disease pathologies. Therefore, our pipeline allows for highly detailed molecular characterization of cell types within the kidney that is key to inform on molecular diagnoses and targeted therapies for kidney diseases.

While renal lineages were well represented in our data, we did find limited coverage of immune cell populations outside the tissue-resident macrophages (Supplementary Fig. 4c). This may represent technical differences associated with whole cell or nuclear dissociation strategies; differences in transcript detection levels between the nucleus and cytosol; or a limited immunological reactivity in most of our samples as confirmed by pathological examination (Supplementary Data 1). Furthermore, compared to the renal cortex, coverage of medullary cell types was from fewer individuals of differing sampling depths (Supplementary Fig. 5d, e). Sampling of more individuals and lower anatomical structures could improve confidence in these identified clusters and permit detection of missing cell populations that include the inner medullary collecting duct cells. Increased sampling and three-dimensional molecular imaging will also better inform on classifications for subsegments of the TL and cortical collecting system that were difficult to assign based solely on existing data from lower mammals.

Our study provides an initial step towards the creation of a molecular and physiological atlas that portrays anatomical nephron organization and that can serve as an important reference to identify deviations in various kidney diseases. Efficacy as a normal reference, however, is limited by underlying diseases or comorbidities often found in adult human specimens, as seen for samples used in this study (Supplementary Data 1). Currently, there is no readily available normal kidney tissue source that can be ethically obtained without harvesting artifacts. Our approach allows correlating pathology, preanalytical parameters and quality control to identify sources of artifacts to best create a reference associated with clinical and histopathological parameters (Supplementary Fig. 12**)**. This is demonstrated by a high consistency observed across individuals and tissue sources (Supplementary Fig. 6), detection of known markers in the appropriate cell types, and validation of known and newly discovered markers using protein analyses. Furthermore, by using an average of 6 mm^3^ of kidney tissue in our assays (Supplementary Data 2), we ensured compatibility with clinically relevant biopsy samples, allowing parallel snRNA-seq, histological/pathological and in situ validations. Therefore, we demonstrate a valuable pipeline for cell-type interrogation of limiting solid tissue samples that can glean molecular insight into normal and pathologic functions.

## Methods

### Human samples and data

All human samples and data were collected by the Kidney Translational Research Center (KTRC) for this study under a protocol approved by the Washington University Institutional Review Board. Informed consent was obtained for the use of data and samples for all participants at Washington University and include living patients undergoing partial or total nephrectomy or from discarded deceased kidney donors (Supplementary Data 1). Samples from University of Michigan were obtained from tumor nephrectomies harvested from consented patients by the Tissue Procurement Service as a part of the Kidney Precision Medicine Project (KPMP) consortium (https://kpmp.org/) and were approved as exempted by the University of Michigan Institutional Review Board because they were anonymized. We have complied with all relevant ethical regulations related to this study. All samples were dissected from tumor-free regions and the composition and pathology was histologically confirmed (Supplementary Data 1). In cases where both cortex and medulla were present (total nephrectomies and deceased donor nephrectomies), cortex was coarsely dissected at the grossly visible boundary between the medulla and the cortex, identified as the junction between the broad base of the pyramid abutting the cortex with often visible lumen of the arcuate arteries at the corticomedullary junction (CMJ).

### Sample processing and nuclei preparation

Dissected tissue was embedded in a cryomold in optimal cutting temperature compound (O.C.T.), frozen on dry ice and stored at −80 °C until further processing (Supplementary Fig. 12 and https://kpmp.org/resources/). For nuclei preparation, 7 × 40 µm-thick sections were collected in the indicated processing solution and used for nuclei isolation. Since about 2-mm-thick tissue pieces were embedded in these blocks, the exact content of cortex varied in grossly dissected cortex at the renal CMJ. Therefore, to confirm tissue composition, 10 µm sections that flank the thick sections used for nuclei isolation were obtained for histology and relative amount of cortex and medulla were determined (Supplementary Data 1). Adjacent formalin or 4% paraformaldehyde fixed, paraffin embedded or O.C.T. cryo-embedded tissue was used for pathological evaluation and immunofluorescence validations, respectively. For fresh tissue dissociation, we applied two-step enzyme treatment protocol modified from Adult mouse DRG dissociation for FACS in GUDMAP Resources (https://www.gudmap.org/chaise/record/#2/Protocol:Protocol/RID=N-H9B0). Briefly, for the first step enzyme treatment, specimens were treated with Papain solution (15 U/ml in Hank’s Balanced Salt Solution with 10 mM 4-(2-hydroxyethyl)-1-piperazineethanesulfonic acid, HEPES) or Trypsin solution (0.05% Trypsin/ 0.02% ethylenediaminetetraacetic acid, EDTA) for 10 or 20 min at 37 °C, followed by mild trituration with P-1000 pipette tip with cut-end, and further treated with Collagenase solution (1.6 mg/ml of collagenase (Catalog number C0130-100MG, batch number SLBS9882, Sigma) in DMEM/F12 with 10% FCS) for 20 min or 40 min at 37 °C for the second step enzyme treatment. Two hundred micrograms of DNaseI was added during Collagenase treatment to keep the cells dissociated and avoid making clumps. After the enzyme treatment, the specimens were partially dissociated by trituration with P-1000 pipette tip with cut-end, then with regular tip, and stored in RNAlater at −20 °C until nuclei were isolated.

Nuclei were prepared from cells or tissues using nuclear extraction buffer (NEB) using established methods^7,8^ with modifications for processing kidney tissue samples, and are outlined below. A step-by-step protocol is also available at https://kpmp.org/resources/. Tissue preparation for nuclei extraction: for whole tissue samples, frozen kidney tissue was chopped briefly on ice; for cryosections, storage buffers (if applicable) were removed, 1 ml NEB (containing 0.1% RNase Inhibitor) was added and initial dissociation of the sections was performed by pipetting up and down using a p1000; for dissociated cells stored in RNAlater, cells were centrifuged at 3000 × g for 5 min, RNAlater was removed, NEB (containing 0.1% RNase Inhibitor) was added to the cells or tissue. Nuclei isolation: nuclei were then extracted on ice using a glass dounce homogenizer (Sigma) using 15−20 up-and-down strokes in 1 ml of NEB. Samples were passed through a 30 µm filter (Sysmex Partec) to remove undissociated tissues, incubated on ice for 0−10 min before washing in PBS + 2 mM EGTA (PBSE) and centrifuged at 900 × g for 5 min at 4 °C. Nuclei pellets were then resuspended in PBSE supplemented with 1% fatty-acid free bovine serum albumin (FAF-BSA, Gemini) containing 4′,6-diamidino-2-phenylindole (DAPI) and either flow sorted (DAPI + singlets using BD Influx, Human Embryonic Stem Cell Core at Sanford Consortium for Regenerative Medicine), used directly for droplet encapsulation, or stored in RNAlater at −20 °C for future processing.

### snDrop-seq library preparation, sequencing and analysis

snDrop-seq, from library preparation through to mapping and digital expression matrix generation of unique molecular identifier (UMI) counts for all genes and all cell barcodes, was performed according to the Drop-seq protocol^47^ (http://mccarrolllab.com/wp-content/uploads/2016/03/Drop-seqAlignmentCookbookv1.2Jan2016.pdf), with modifications^8^ and are provided as supplementary software: https://github.com/chensong611/Dropseq_pipeline. The step-by-step protocol is also available at https://kpmp.org/resources/. Primers used in this protocol are listed in Supplementary Data 15. Briefly, following encapsulation of the nuclei, droplets were overlaid with a layer of mineral oil; incubated at 72 °C (water bath) for 5 min; transferred to ice and droplets broken by perfluorooctanol. Beads were then harvested; hybridized RNA was reverse-transcribed; and cDNA was PCR amplified for 16 cycles as described^47^. cDNA from each replicate library (Supplementary Data 2) was then tagmented using Nextera XT using distinct Nextera index 1 primers, pooled and sequenced on an Illumina HiSeq 2500 with Read1CustSeqB^47^ for priming of read 1 (read 1 was 30 bp; read 2 (paired end) was 80 bp). Each cell barcode was tagged by an associated library batch ID (Supplementary Data 2), count matrices were combined across experiments and primary QC filters were applied (Supplementary Fig. 2): only cell barcodes having >400 nonmitochondrial transcripts were included; transcripts detected in <3 cells or that were mitochondrially expressed were excluded; and cell barcodes that detected >400 and <5000 genes were kept for downstream analyses. Secondary QC filters and clustering analysis was performed using PAGODA2 ^8^ (https://github.com/hms-dbmi/pagoda2): a gene/UMI ratio filter was applied to remove low-quality nuclei that deviated from the group trend (confidence interval < 1E-10), counts were then normalized to the total number of counts for each nucleus, and batch variations were corrected by scaling expression of each gene such that the within batch expression average matched the dataset-wide average. After variance normalization, the top 2000 overdispersed genes were identified and used for principal component analysis. Clustering was performed using an approximate *k*-nearest neighbor graph (*k* = 30) based on a cosine distance of the top 150 principal components and cluster identities determined using the infomap community detection algorithm. Sparsely populated clusters tend to represent low-quality data and possible multiplets (typically occurring as outliers to the main cell-type clusters when visualized using T-distributed Stochastic Neighbor Embedding or t-SNE). As such, poorly clustered nuclei that formed clusters of less than 30 were excluded and an additional round of clustering was performed. Again clusters having less than 30 nuclei were excluded to further remove low-quality data and doublets. To assess stability of the clusters and the appropriateness of the *k* = 30 value used, cluster identities were determined using the infomap community detection algorithm from a series of *k* values (10, 20, 30, 40, 50) and Jaccard similarity was calculated using the compare_annotate() function of the scrattch.hicat R package (https://github.com/AllenInstitute/scrattch.hicat). For cluster visualization, uniform manifold approximation and projection (UMAP) dimensional reduction was performed in Seurat (version 2.3.4) using the top 150 principal components identified using PAGODA2. One cluster of 120 nuclei failed to show distinct marker gene expression (no genes showing within-cluster average gene expression values >1 log fold change over averaged expression across remaining clusters) and so was excluded as possible low-quality data. Remaining clusters were manually annotated based on known cell-type marker genes (Supplementary Data 5). Subsequent expression analyses (dot plots, violin plots, expression heatmaps, feature plots) were performed using Seurat software (version 2.3.4, https://satijalab.org/seurat/) where counts for all cell barcodes used in PAGODA2 clustering analysis were scaled by total UMI counts, multiplied by 10,000 and transformed to log space. Technical effects of batch and UMI coverage were regressed from scaled data using the RegressOut function (Seurat). Differential expression analysis between clusters was performed using a Wilcoxon rank sum test (Seurat) on all genes detected in at least 25% of nuclei within a cluster. Marker gene selection criteria for plots generated in this manuscript: Figs. 2a, 3d—manually curated known marker genes (Supplementary Data 5) and cluster-enriched marker genes (Supplementary Data 7); Figs. 4b, c, 5b, f—manually curated known marker genes (Supplementary Data 5), marker genes identified from differential expression analysis of all clusters (Supplementary Data 7) and marker genes identified from differential expression analysis of cluster subsets (Supplementary Data 11−14); Supplementary Figs. 2b, c, 9c, 10b—as specified in the figures and derived from Supplementary Data 7, 11, 12, 13, 14; Supplementary Fig. 8c—as specified in the figures and derived from Supplementary Data 5 and 7. For correlation analyses between different experimental conditions or individuals, averaged gene expression values were calculated on raw counts in Seurat and Pearson correlation coefficients visualized using the corrplot package in R.

For pairwise correlation of clusters and cluster association analysis, averaged expression values for each cluster were calculated on scaled expression values for the top 2000 variable genes identified using PAGODA2. A correlation heatmap was generated using the corrplot package with sample ordering based on the hierarchical clustering method ward.D. A cluster association graph was generated from the cluster pairwise correlation matrix and visualized by the Fruchterman−Reingold layout algorithm using the igraph package in R. Cluster dot size was scaled to reflect the associated number of nuclei making up that cluster.

For integrin enrichment analysis, integrin genes were manually curated, and averaged expression values were calculated for each cluster using Seurat. Genes showing significant differential expression between clusters (adjusted *p* value < 0.05) were identified using the Wilcoxon rank sum test (Seurat), duplicated genes were kept only for the cluster having the highest log fold change, and heatmaps of averaged expression values were plotted using the gplots package in R.

### SWNE and trajectory analyses

SWNE analysis^26^ was performed (https://yanwu2014.github.io/swne/) on the indicated cluster subsets using the pre-computed Pagoda2 object outlined above, with variant genes and principal components from each cluster subset recalculated independently using Pagoda2 (as described above) and nonnegative matrix factorization (NMF) run using the “ica” initialization method. SWNE embedding was calculated using the following parameters: alpha.exp = 1.8, snn.exp = 0.1, n_pull = 6, dist.use = “cosine” (or “pearson” for nephron/CD combined clusters). For trajectory analysis using Monocle (Version 2.6.4), analysis was performed in R according to the provided documentation (http://cole-trapnell-lab.github.io/monocle-release/), and with UMI counts modeled as a negative binomial distribution. Ordering genes were determined as the top 500 differentially expressed genes (expressed in at least ten nuclei) that were identified using the differentialGeneTest function (Monocle). Reduction to two dimensions was performed using the discriminative dimensionality reduction with trees (DDRTree) method. Genes showing significant variation by pseudotime (*q* value < 0.01) were identified and plotted using the plot_pseudotime_heatmap function (Monocle). Gene sets (Supplementary Data 10) were identified by running the plot_pseudotime_heatmap function and setting the number of clusters to three. Gene ontology analysis was performed using the ToppGene Suite (https://toppgene.cchmc.org/).

### Comparison of snDrop-seq data with published data

For comparison with mouse whole-cell data^17^, the associated raw counts were downloaded from the Gene Expression Omnibus (GSE107585) and processed using Seurat: samples having greater than 20% mitochondrial transcripts were excluded; counts for all cell barcodes were scaled by total UMI counts, multiplied by 10,000 and transformed to log space; variable genes were identified using the mean variability plot (x.low.cutoff = 0, x.high.cutoff = 3; y.cutoff = 0.8); technical effects of batch and UMI coverage were regressed from scaled data using the RegressOut function. Averaged scaled gene expression values for each cluster were then calculated (Seurat) using a common set of marker genes (*n* = 262) identified by intersecting the variable genes from the mouse data (*n* = 1460) with those identified by Pagoda2 above (*n* = 2000). For comparison with published human single-nucleus and single-cell data from a single tumor-free nephrectomy sample and a single post-transplant biopsy sample, the associated raw counts were downloaded from the Gene Expression Omnibus (GSE109564 and GSE114156) and cell-type clusters and variable genes were identified using the Seurat parameters that were described for this study^18^: samples having <300 (biopsy) or <400 (nuclei) and >4000 genes detected and having greater than 20% mitochondrial transcripts (biopsy only) were excluded; counts for all cell barcodes were scaled by total UMI counts, multiplied by 10,000 and transformed to log space; variable genes were identified using the mean variability plot (x.low.cutoff = 0.0125, x.high.cutoff = 6 (biopsy) or 3 (nuclei); y.cutoff = 1 (biopsy) or 1.2 (nuclei)); technical effects of mitochondrial transcript percentage (biopsy only) and/or UMI coverage were regressed from scaled data using the RegressOut function; t-SNE was performed on the first 20 (biopsy) or 9 (nuclei) principal components; and clusters identified using the FindClusters function in Seurat (resolution = 1.2 (biopsy) or 0.6 (nuclei)). Average scaled gene expression values for each cluster were calculated using a common set of marker genes (*n* = 332, whole cell versus nuclei; *n* = 346, nuclei versus nuclei) identified by intersecting the variable genes that were used for clustering each of the data sets (2405 for published whole cell; 2247 for published nuclei) with those identified by Pagoda2 above (*n* = 2000). Heatmaps of correlation values were generated using the corrplot package in R. For cluster enrichment analysis for eQTL associated with CKD^21^ and loci associated with blood pressure traits^22^: the associated genes were obtained from published supplementary tables (indicated in Supplementary Data 8); gene symbols were filtered to remove duplicated values; Wilcoxon rank sum tests (Seurat) were performed to identify genes showing significant differential expression between clusters (adjusted *p* value < 0.05, Wilcoxon rank sum test, Supplementary Data 8); and duplicated gene values were kept only for the cluster having the highest log fold change. To determine cluster enrichment, gene specificity scores were calculated on expression values for the identified differentially expressed genes using a method that accounted for inter-cluster differences by weighting for cluster distance metrics^48^ (specificityScore function, https://github.com/Steven-M-Hill/PCHiC-specificity-score-analysis). To assess cluster enrichment for CKD or hypertension risk factors, the proportions of differentially expressed genes identified for each cluster were calculated. Corresponding heatmaps for gene specificity scores and cluster enrichment proportions were plotted using the gplots package in R.

### Protein expression data and immunostaining

Individual protein immunohistochemical stains (Figs. 3e, 4e, 5c, g, 6b, Supplementary Figs. 8a, 10c, 11a) were obtained from The Human Protein Atlas^49^ (http://www.proteinatlas.org) and referenced in Supplementary Data 16. Fluorescent immunostainings (single or combined) were performed on 4% paraformaldehyde-fixed cryopreserved sections (10 µm) and visualized using confocal immunofluorescence microscopy^50,51^. Briefly, slides were dried and post-fixed with 4% paraformaldehyde at room temperature (RT) for 12 min, followed by washing twice in phosphate-buffered saline (PBS). Blocking and permeabilization were with 1% BSA/0.2% skim milk/0.3% Triton X-100 in PBS (PBS-BB) for 1 h at RT followed by overnight incubation at 4 °C with primary antibody diluted in PBS-BB. After the primary antibody incubation, slides were washed three times with 0.3% Triton X-100 in PBS (PBS-Tr) at RT, each for 5 min. After the washing, slides were incubated for 1 h at RT with secondary antibody mixture (1:200 dilution in PBS-BB) containing alexa-488, cy3, or cy5 (or Dyelight-647) (Jackson Immuno Research). Slides were washed once with PBS-Tr at RT for 5 min, followed by staining with bisbenzimide for nuclear staining and finally washing twice with PBS-Tr, before mounting with ProLong Diamond (Thermo Fisher Scientific). Images were obtained with Nikon C1 confocal system and analyzed with Nikon Elements software (Nikon). Full details on the antibodies used are provided in Supplementary Data 17. All image panels were assembled in Adobe Photoshop.

### Reporting summary

Further information on research design is available in the Nature Research Reporting Summary linked to this article.

## Supplementary information

Supplementary Information

Description of Additional Supplementary Files

Supplementary Data 1

Supplementary Data 2

Supplementary Data 3

Supplementary Data 4

Supplementary Data 5

Supplementary Data 6

Supplementary Data 7

Supplementary Data 8

Supplementary Data 9

Supplementary Data 10

Supplementary Data 11

Supplementary Data 12

Supplementary Data 13

Supplementary Data 14

Supplementary Data 15

Supplementary Data 16

Supplementary Data 17

Reporting Summary

Source Data

## Data Availability

Raw snDrop-seq RNA sequencing data and annotated digital expression matrices are available from the NCBI Gene Expression Omnibus, accession code GSE121862. All relevant data are also available from the corresponding authors upon request. Previously published data that was used in this study are also available from NCBI GEO: GSE107585; GSE109564; GSE114156. Source data underlying Fig. 1d, Supplementary Figs. 1a, 1b, 5e, 6b are provided as Source Data File 1. Source data underlying Figs. 1b, 2, 3a−d, 3f, 4a−d, 5a, b, 5e, f, 5h, 6a and Supplementary Figs. 2−7, 8b, c, 9, 10b, 11 are provided as Source Data File 2. Additional phenotyping data on participants PPID 3351, 3395, 3411, 3412, 3414, 3431, 3432, 3434, 3435, 3444 are available upon reasonable request to sanjayjain@wustl.edu.
