# Supplementary material for: A single-nucleus RNA-sequencing pipeline to decipher the molecular anatomy and pathophysiology of human kidneys
Source: Nat Commun. 2019 Jun 27;10:2832. doi: 10.1038/s41467-019-10861-2 (PMC6597610; doi:10.1038/s41467-019-10861-2)
Supplement: Supplementary file 1 — Supplementary Information [file 41467_2019_10861_MOESM1_ESM.pdf]

SUPPLEMENTARY INFORMATION

A single-nucleus RNA-sequencing pipeline to decipher the molecular anatomy and pathophysiology of human kidneys

Lake et al.

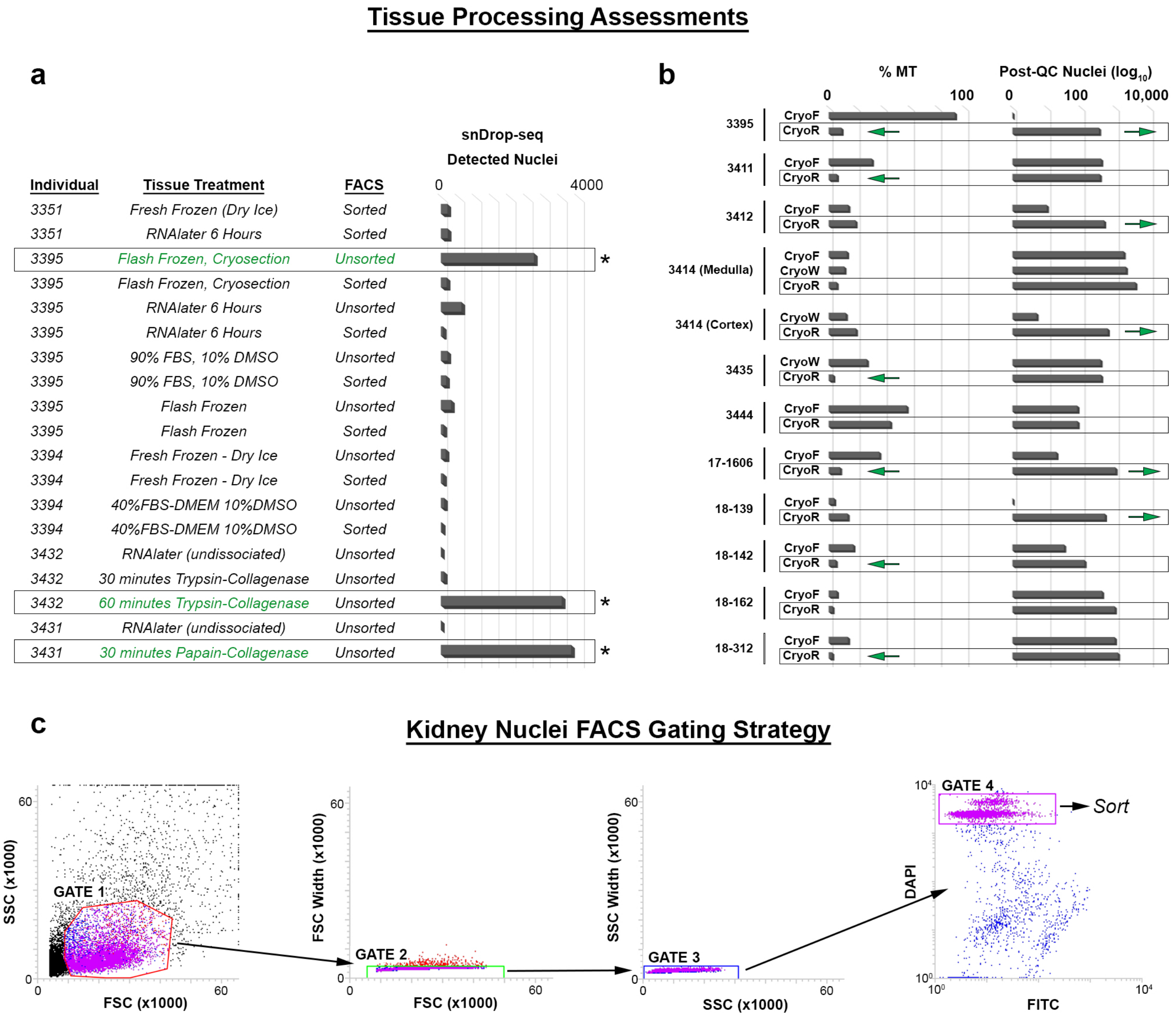

Supplementary Figure 1. Adult human kidney tissue processing assessment. a. Nuclei from different tissue preservation and processing methods within and across individuals (n = 5) were processed by snDrop-seq and the number of nuclei (> 400 UMI detected) relative to total number of nuclei loaded is shown. Asterisks indicate conditions showing positive outcome. **b.** Comparison of snDrop-seq outcome using nuclei isolated from cryosections from the same samples (n = 12) that were treated in different ways (cryoF, cryoW, cryoR). Percent MT (nuclei >400 UMI) and number of post-QC nuclei (nuclei > 400 nonMT UMI, >400 and < 5000 genes detected) are shown. Arrows indicate lower percent MT and higher post-QC yields for cryoR samples. **c.** FACS gating strategies for DAPI stained kidney nuclei used in (**a**). Source data for (**a**) and (**b**) are provided as a Source Data File.

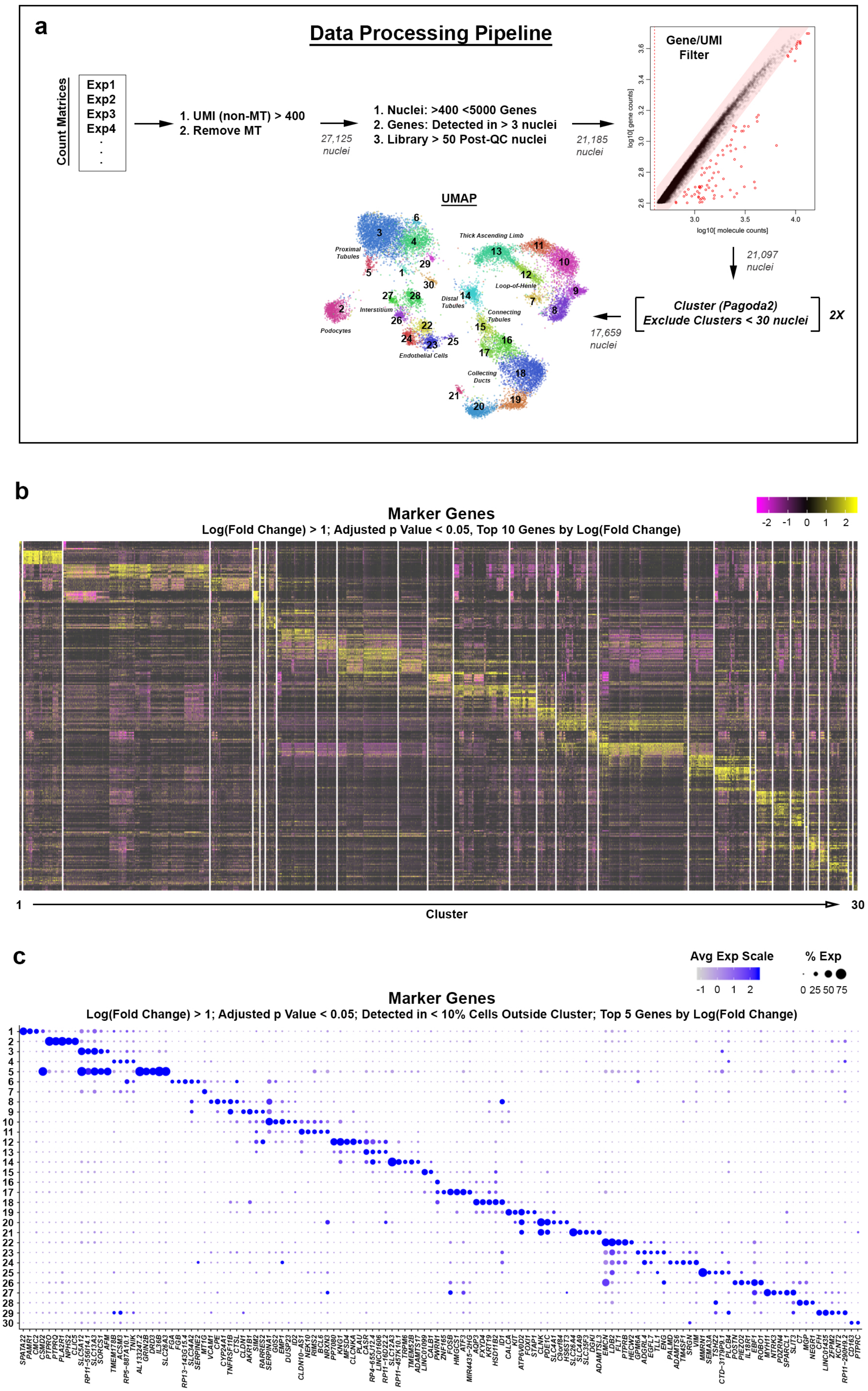
a
Data Processing Pipeline
Count Matrices
Exp1
Exp2
Exp3
Exp4
1. UMI (non-MT) > 400
2. Remove MT
27,125 nuclei
1. Nuclei: >400 <5000 Genes
2. Genes: Detected in > 3 nuclei
3. Library > 50 Post-QC nuclei
21,185 nuclei
Gene/UMI Filter
log10[gene counts]
log10[molecule counts]
21,097 nuclei
Cluster (Pagoda2)
Exclude Clusters < 30 nuclei
2X
17,659 nuclei
UMAP
Proximal Tubules
Thick Ascending Limb
Loop-of-Henle
Distal Tubules
Connecting Tubules
Interstitium
Podocytes
Endothelial Cells
Collecting Ducts
b
Marker Genes
Log(Fold Change) > 1; Adjusted p Value < 0.05, Top 10 Genes by Log(Fold Change)
Cluster
c
Avg Exp Scale
% Exp
Marker Genes
Log(Fold Change) > 1; Adjusted p Value < 0.05; Detected in < 10% Cells Outside Cluster; Top 5 Genes by Log(Fold Change)

Supplementary Figure 2. Single nucleus interrogation of the human kidney. a. Data processing pipeline. Count matrices across experiments were combined and only nuclei detecting more than 400 non-MT UMI were included. Mitochondrial genes not expressed in the nucleus were excluded. Nuclei detecting more than 400 and less than 5000 genes were then subjected to a gene/UMI ratio filter (see Methods) to further remove low quality data prior to clustering using Pagoda2 software. Two rounds of clustering were performed to remove nuclei that failed to cluster or that formed clusters of less than 30 nuclei. This allowed further removal of low-quality nuclei or multiplets. Remaining clusters that were derived from the 17,659 post-QC nuclei were visualized by UMAP. The number of nuclei after each stage of processing are shown. **b.** Heatmap of expression of marker genes defining clusters shown in **Fig. 1b** using criteria indicated and for differentially expressed genes found in **Supplementary Data 7**. **c.** Dot plot of marker gene expression values (log scale) and percentage of nuclei expressing these genes within each cluster (**Fig. 1b**) using criteria indicated and for differentially expressed genes found in **Supplementary Data 7.**

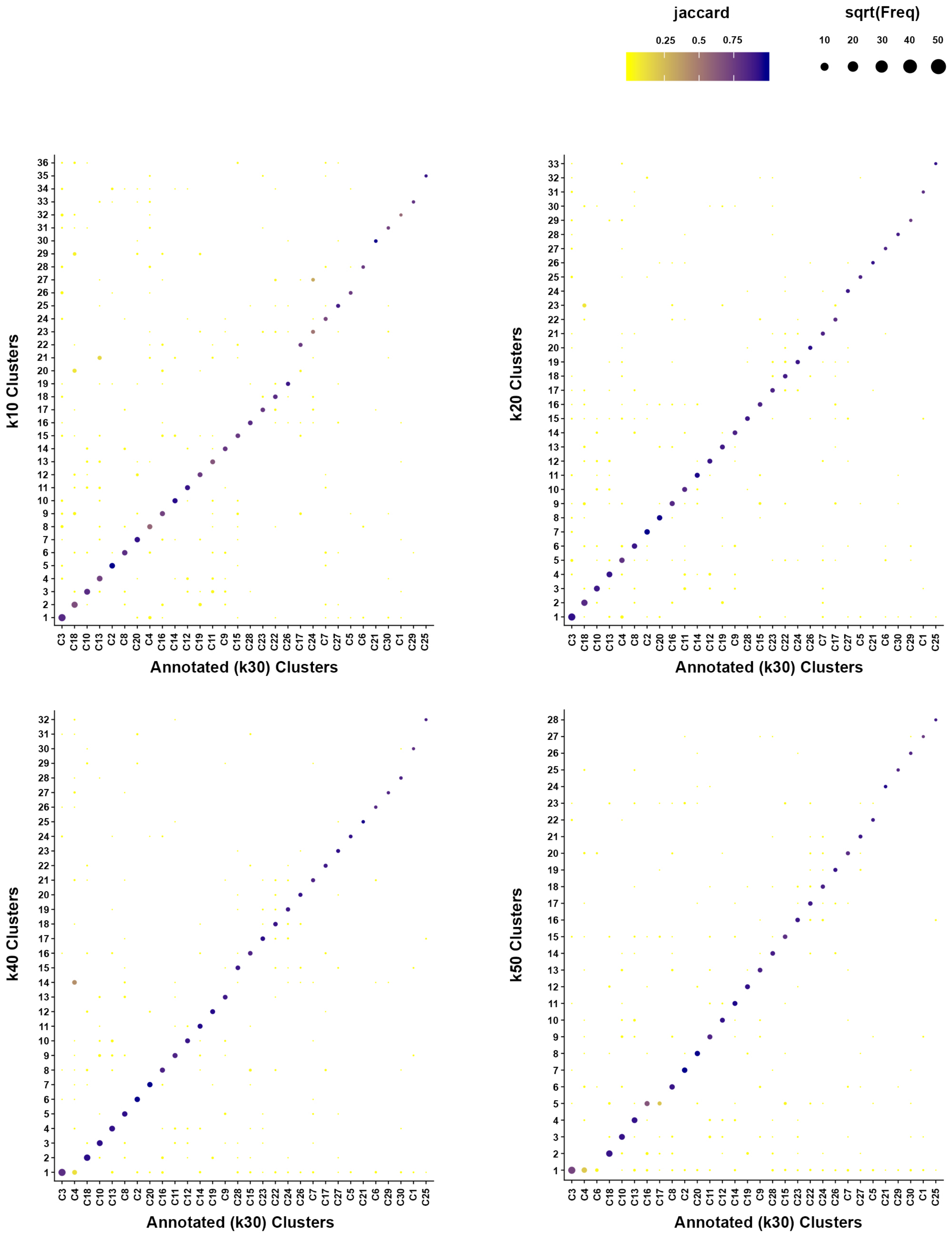
jaccard
0.25 0.5 0.75
sqrt(Freq)
10 20 30 40 50
k10 Clusters
Annotated (k30) Clusters
k20 Clusters
Annotated (k30) Clusters
k40 Clusters
Annotated (k30) Clusters
k50 Clusters
Annotated (k30) Clusters

Supplementary Figure 3. Stability of single nucleus clusters. Cluster identities derived using different k values were compared against the final cluster identities shown in **Fig.1b** using jaccard similarity index to indicate extent of overlap for the single nuclei barcodes (see Methods). For the most part nuclei were similarly clustered at differing k values, with a few smaller sub-clusters arising at lower k values (e.g. C18 or PC-3 splitting into two additional small sub-clusters 20 and 29 at k = 10), and a few clusters merging at high k values (e.g. PT clusters C3, C4 and C6 merging into a single PT cluster at k = 50).

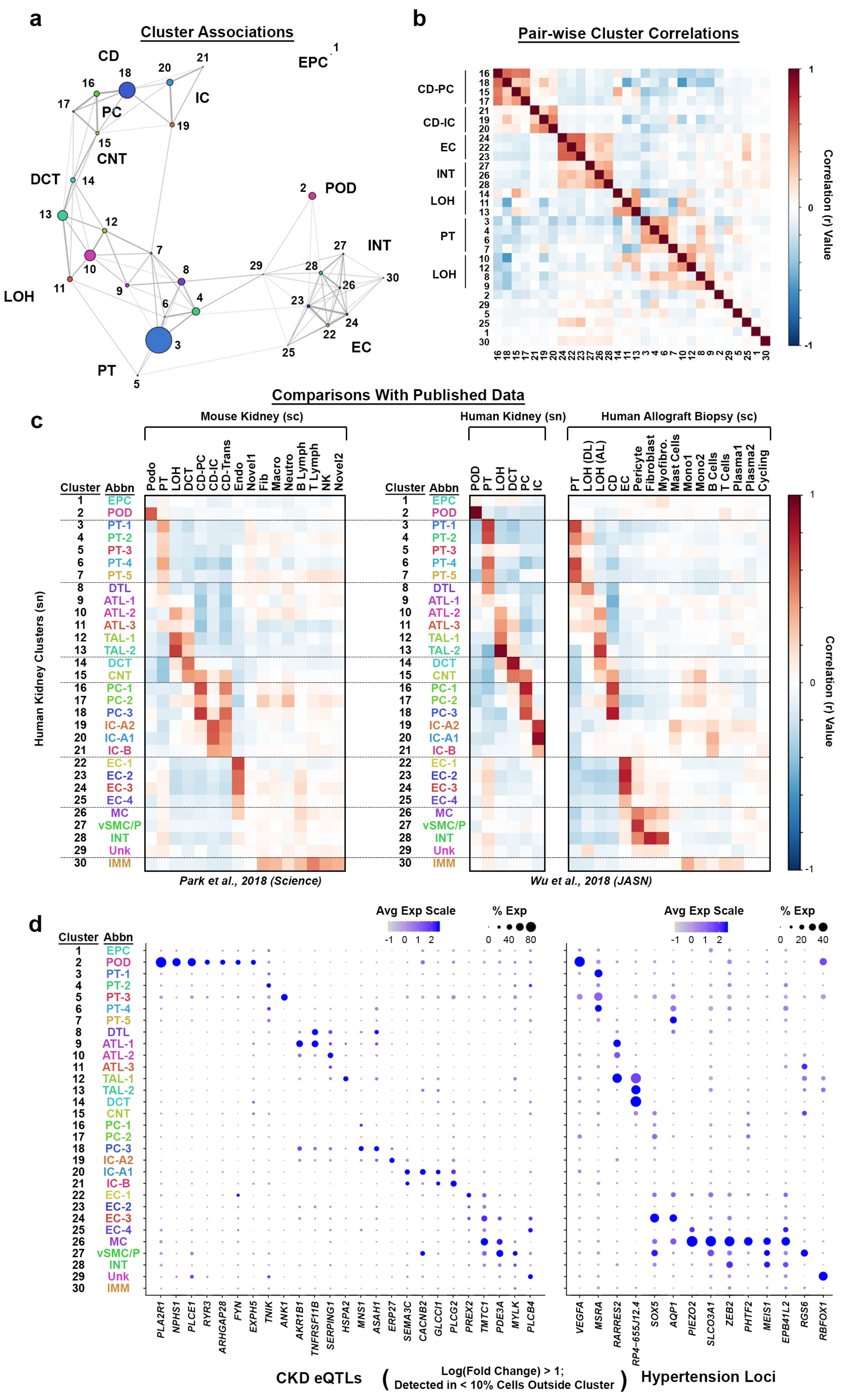

a
Cluster Associations
CD
PC
IC
CNT
DCT
LOH
PT
POD
INT
EC
EPC
b
Pair-wise Cluster Correlations
CD-PC
CD-IC
EC
INT
LOH
PT
LOH
Correlation (r) Value
Comparisons With Published Data
c
Mouse Kidney (sc)
Human Kidney (sn)
Human Allograft Biopsy (sc)
Cluster
Abbn
Podo
PT
LOH
DCT
CD-PC
CD-IC
CD-Trans
Endo
Novel1
Fib
Macro
Neutro
B Lymph
T Lymph
NK
Novel2
POD
PT
LOH
DCT
PC
IC
PT
LOH (DL)
LOH (AL)
CD
EC
Pericyte
Fibroblast
Myofibro.
Mast Cells
Mono1
Mono2
B Cells
T Cells
Plasma1
Plasma2
Cycling
1 EPC
2 POD
3 PT-1
4 PT-2
5 PT-3
6 PT-4
7 PT-5
8 DTL
9 ATL-1
10 ATL-2
11 ATL-3
12 TAL-1
13 TAL-2
14 DCT
15 CNT
16 PC-1
17 PC-2
18 PC-3
19 IC-A2
20 IC-A1
21 IC-B
22 EC-1
23 EC-2
24 EC-3
25 EC-4
26 MC
27 vSMC/P
28 INT
29 Unk
30 IMM
Human Kidney Clusters (sn)
Park et al., 2018 (Science)
Wu et al., 2018 (JASN)
Correlation (r) Value
d
Avg Exp Scale
% Exp
PLA2R1
NPHS1
PLCE1
RYR3
ARHGAP28
FYN
EXPH5
TNIK
ANK1
AKR1B1
TNFRSF11B
SERPING1
HSPA2
MNS1
ASAH1
ERP27
SEMA3C
CACNB2
GLCCI1
PLCG2
PREX2
TMTC1
PDE3A
MYLK
PLCB4
VEGFA
MSRA
RARRES2
RP4−655J12.4
SOX5
AQP1
PIEZO2
SLCO3A1
ZEB2
PHTF2
MEIS1
EPB41L2
RGS6
RBFOX1
CKD eQTLs
Log(Fold Change) > 1;
Detected in < 10% Cells Outside Cluster
Hypertension Loci

Supplementary Figure 4. Distinct cell types resolved from single nucleus data. a. A cluster association plot where thickness of connecting lines indicates relative correlation of averaged scaled expression values for the top 2000 variant genes used to define clusters, and point size indicates number of nuclei within each cluster. **b.** Hierarchical clustering (ward.D method) heatmap for pairwise correlation values that were generated on averaged scaled expression values for the top 2000 variant genes used for PAGODA2 clustering. **c.** Clusters shown in **(a)** were compared with published single-cell or single-nucleus data from the mouse or human kidney. Heatmaps show correlation values for cluster-averaged scaled expression values for shared top variant genes detected in each data set (see Methods). **d.** Dot plot of a subset of distinct CKD-associated eQTLs and hypertension risk loci expression values (log scale), and percentage of nuclei expressing these genes within each cluster (**Fig. 1b**), using the selection criteria indicated and for differentially expressed genes found in **Supplementary Data 8.**

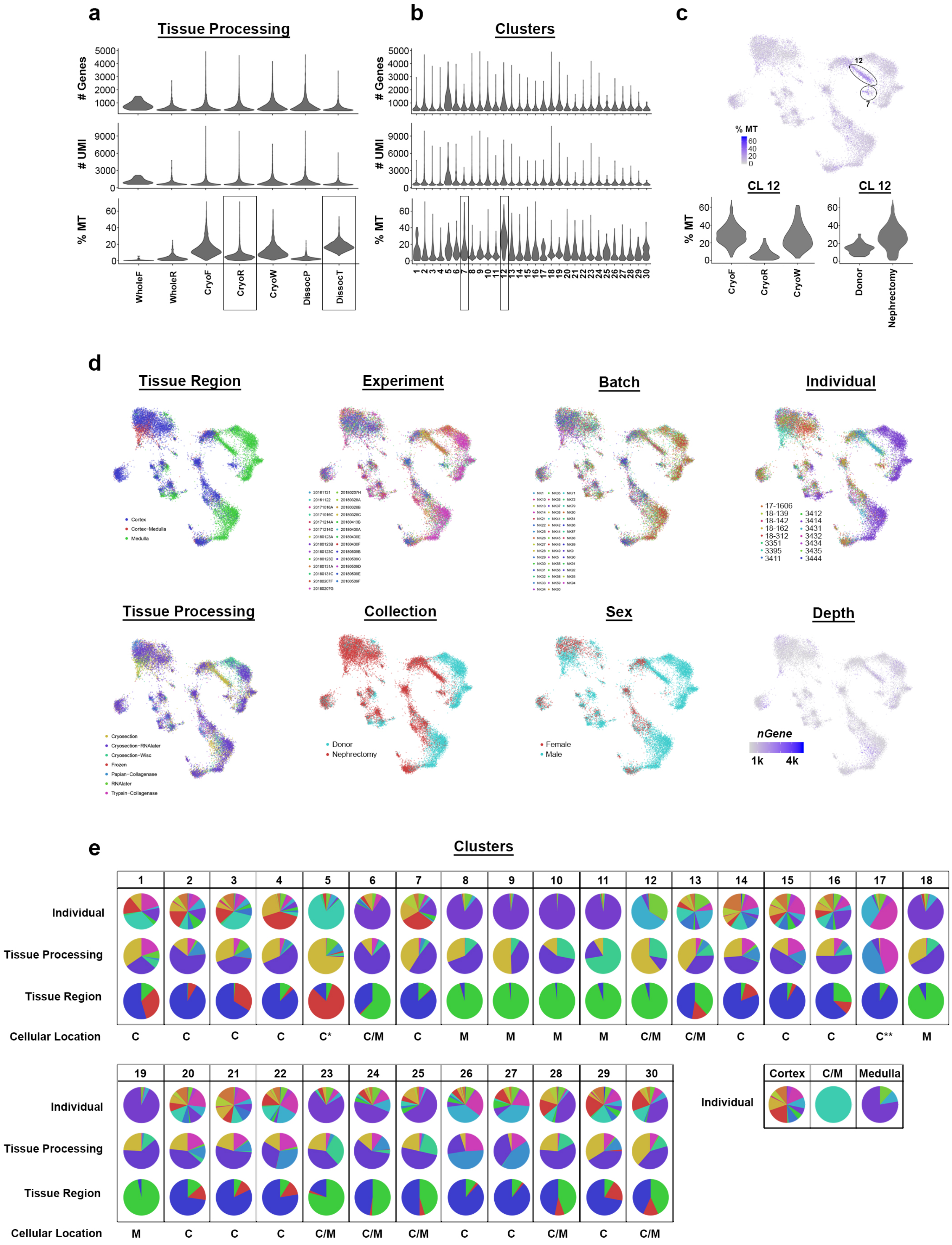
a
Tissue Processing
Genes
UMI
% MT
WholeF
WholeR
CryoF
CryoR
CryoW
DissocP
DissocT
b
Clusters
c
% MT
CL 12
CryoF
CryoR
CryoW
Donor
Nephrectomy
d
Tissue Region
Cortex
Cortex−Medulla
Medulla
Experiment
Batch
Individual
Tissue Processing
Cryosection
Cryosection−RNAlater
Cryosection−Wisc
Frozen
Papian−Collagenase
RNAlater
Trypsin−Collagenase
Collection
Donor
Nephrectomy
Sex
Female
Male
Depth
nGene
1k
4k
e
Clusters
Individual
Tissue Processing
Tissue Region
Cellular Location
C C C C C* C/M C M M M M C/M C/M C C C C** M
M C C C C/M C/M C/M C C C/M C C/M
Cortex
C/M
Medulla

Supplementary Figure 5. QC assessment of snDrop-seq data. a. Violin plots indicating number of genes and UMI detected per nucleus and the percent MT that were detected for 17,659 post-QC nuclei (**Supplementary Data 4**) grouped by tissue processing conditions. **b.** Similar metrics as in (**a**) except for nuclei grouped by cluster identity. Boxes indicate S3 PT (PT-5, cluster 7) and TAL (TAL-1, cluster 12) clusters showing elevated %MT. **c.** UMAP plot as shown in **Fig. 1b** showing percent MT across single nuclei. Clusters highlighted in **(b)** are indicated. Violin plots show percent MT for cluster 12 (TAL-1) nuclei only grouped by either tissue processing or tissue procurement method. **d.** UMAP plots as shown in **Fig. 1b** showing different metadata components associated with each nucleus (**Supplementary Data 4)**, including: tissue region, experiment, batch (library), individual, tissue processing method used, collection or procurement method, sex and gene depth. **e.** Pie charts for each cluster showing proportions contributed from different individuals, different tissue processing methods and different tissue regions. Predicted origin for each cluster to the cortex or medulla is indicated. *Predicted artifactual cortical PT cluster derived mostly from a single individual. **Predicted artifactual CD cluster derived primarily from tissue processing methods involving enzymatic dissociation (dissocPC and dissocTC). Source data for (**e**) is provided as a Source Data File.

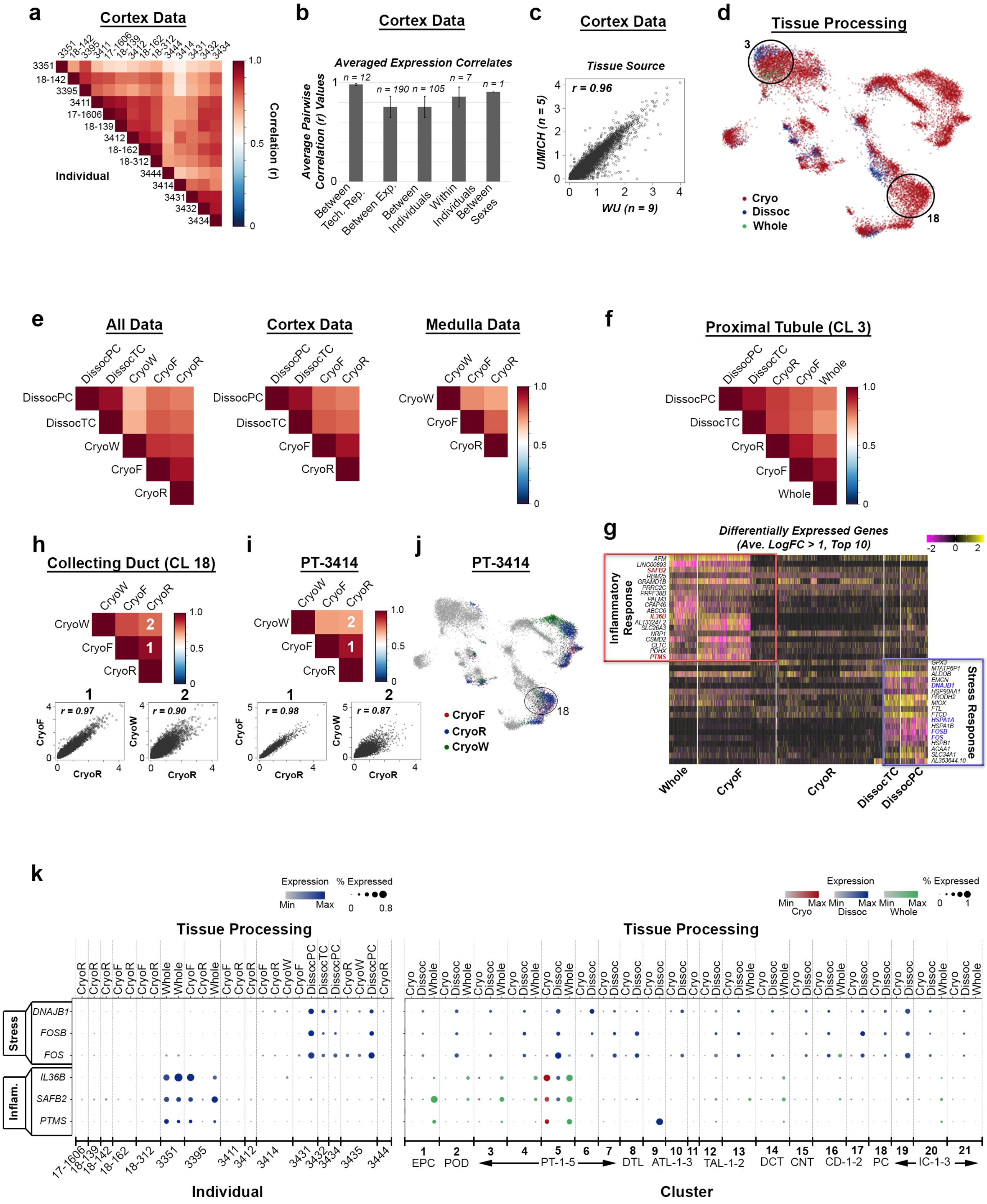
a
Cortex Data
Individual
Correlation (r)
b
Cortex Data
Averaged Expression Correlates
Average Pairwise Correlation (r) Values
n = 12
n = 190
n = 105
n = 7
n = 1
Between Tech. Rep.
Between Exp.
Between Individuals
Within Individuals
Between Sexes
c
Cortex Data
Tissue Source
r = 0.96
UMICH (n = 5)
WU (n = 9)
d
Tissue Processing
Cryo
Dissoc
Whole
e
All Data
Cortex Data
Medulla Data
DissocPC
DissocTC
CryoW
CryoF
CryoR
f
Proximal Tubule (CL 3)
Whole
g
Differentially Expressed Genes
(Ave. LogFC > 1, Top 10)
Inflammatory Response
Stress Response
h
Collecting Duct (CL 18)
r = 0.97
r = 0.90
i
PT-3414
r = 0.98
r = 0.87
j
PT-3414
CryoF
CryoR
CryoW
k
Expression
% Expressed
Tissue Processing
Stress
Inflam.
DNAJB1
FOSB
FOS
IL36B
SAFB2
PTMS
Individual
Cluster
EPC
POD
PT-1-5
DTL
ATL-1-3
TAL-1-2
DCT
CNT
CD-1-2
PC
IC-1-3

Supplementary Figure 6. QC and reproducibility assessment across individuals and tissue processing methods. a. Heatmap of Pearson correlation values (r) that were generated on averaged expression values (all genes) for nuclei grouped by the different individuals they were isolated from (renal cortex data only, 14 individuals). **b.** Bar chart of averaged correlation values generated on nuclei (cortex data only) grouped by the conditions indicated. Correlations were based on average expression values (all genes), *n* refers to the number of comparisons performed, and error bars indicate standard deviation (sd) between comparisons. Source data is provided as a Source Data File. **c.** Scatter plot for averaged gene expression values (log transformed) for nuclei grouped by tissue source. Associated correlation value and number of individuals (cortex only) the nuclei were derived from are indicated. **d.** UMAP as shown in **Fig. 1b** showing general tissue processing methods used. PT cluster 3 and CD PC cluster 18, used in subsequent analyses, are indicated. **e.** Correlation heatmaps for averaged expression of all genes separately performed on all nuclei, cortex-only nuclei or medulla-only nuclei that were grouped by tissue processing method. **f.** Correlation heatmap for averaged expression of all genes performed on PT cluster 3 nuclei grouped by tissue processing method. **g.** Heatmap of differentially expressed genes identified between tissue processing methods (average log(fold-change) > 1, top 10 genes shown) for PT cluster 3. Gene categories (Inflammatory Response and Stress Response) were based on highlighted genes. **h.** Correlation heatmap for averaged expression of all genes performed on CD cluster 18 nuclei grouped by tissue processing method. Representative scatter plots for comparisons indicated are shown. **i.** Correlation heatmap for averaged expression of all genes performed on nuclei from a single individual (patient 3414) grouped by tissue processing method. Representative scatter plots for comparisons indicated are shown. **j.** UMAP as shown in **Fig. 1b** showing general tissue processing methods used specifically for patient 3414. Cluster 18 is indicated. **k.** Dot plot showing gene expression and associated detection rates for genes highlighted in **(g)** for all 17,659 post-QC nuclei grouped by the conditions indicated. Stress response genes show as specific for dissocPC/TC and across all clusters, indicating a general tissue processing effect. Inflammatory response genes associate with specific individuals (3351/3395) and were found more in PT clusters, likely reflecting an individual-related artifact rather than a tissue processing effect.

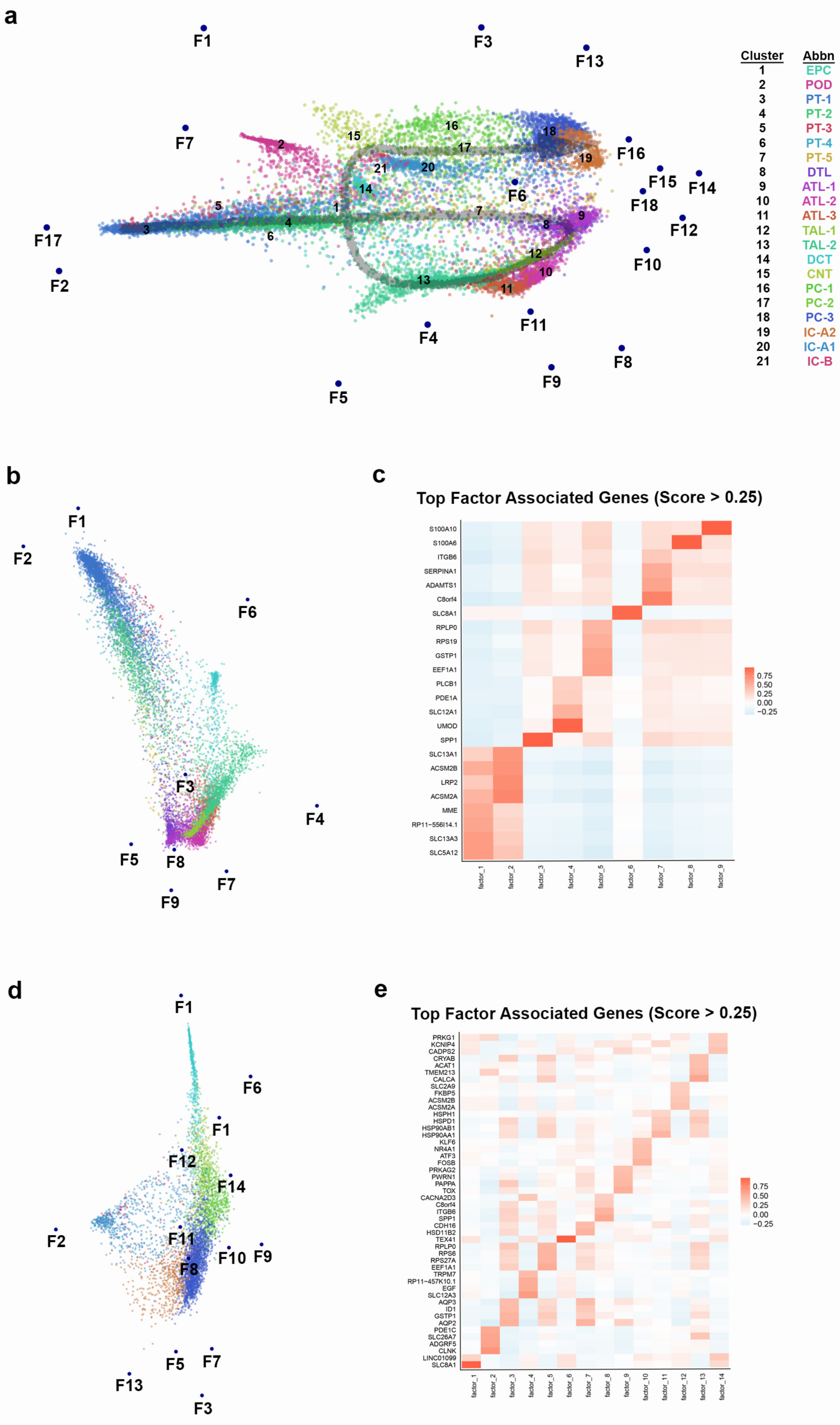
a
F1
F3
F13
F7
F16
F15
F14
F6
F18
F12
F17
F10
F2
F4
F11
F8
F9
F5
Cluster
Abbn
1 EPC
2 POD
3 PT-1
4 PT-2
5 PT-3
6 PT-4
7 PT-5
8 DTL
9 ATL-1
10 ATL-2
11 ATL-3
12 TAL-1
13 TAL-2
14 DCT
15 CNT
16 PC-1
17 PC-2
18 PC-3
19 IC-A2
20 IC-A1
21 IC-B
b
F1
F2
F6
F3
F4
F5
F8
F7
F9
c
Top Factor Associated Genes (Score > 0.25)
S100A10
S100A6
ITGB6
SERPINA1
ADAMTS1
C8orf4
SLC8A1
RPLP0
RPS19
GSTP1
EEF1A1
PLCB1
PDE1A
SLC12A1
UMOD
SPP1
SLC13A1
ACSM2B
LRP2
ACSM2A
MME
RP11−556I14.1
SLC13A3
SLC5A12
factor_1
factor_2
factor_3
factor_4
factor_5
factor_6
factor_7
factor_8
factor_9
0.75
0.50
0.25
0.00
−0.25
d
F1
F6
F1
F12
F14
F2
F11
F10
F9
F8
F5
F7
F13
F3
e
Top Factor Associated Genes (Score > 0.25)
PRKG1
KCNIP4
CADPS2
CRYAB
ACAT1
TMEM213
CALCA
SLC2A9
FKBP5
ACSM2B
ACSM2A
HSPH1
HSPD1
HSP90AB1
HSP90AA1
KLF6
NR4A1
ATF3
FOSB
PRKAG2
PWRN1
PAPPA
TOX
CACNA2D3
C8orf4
ITGB6
SPP1
CDH16
HSD11B2
TEX41
RPLP0
RPS6
RPS27A
EEF1A1
TRPM7
RP11−457K10.1
EGF
SLC12A3
AQP3
ID1
GSTP1
AQP2
PDE1C
SLC26A7
ADGRF5
CLNK
LINC01099
SLC8A1
factor_1
factor_2
factor_3
factor_4
factor_5
factor_6
factor_7
factor_8
factor_9
factor_10
factor_11
factor_12
factor_13
factor_14
0.75
0.50
0.25
0.00
−0.25

Supplementary Figure 7. SWNE analyses. a. Nephron and collecting duct cell populations (clusters 1-21) were visualized using SWNE (see Methods). Arrow indicates expected tubular progression of cell types seen *in vivo*. NMF factors (F1-F18) driving the spatial distribution of the nuclei are indicated. **b.** Spatial distribution of renal tubule cell populations (clusters 2-14) visualized using SWNE. Associated NMF factors (F1-F9) are shown. **c.** Heatmap of the top genes associated with factors shown in **(b)**. **d.** Spatial distribution of distal tubules through collecting duct cell populations (clusters 14-21) visualized using SWNE. Associated NMF factors (F1-F14) are shown. **e.** Heatmap of the top genes associated with factors shown in **(d)**. All factor associated genes can be found in **Supplementary Data 9**.

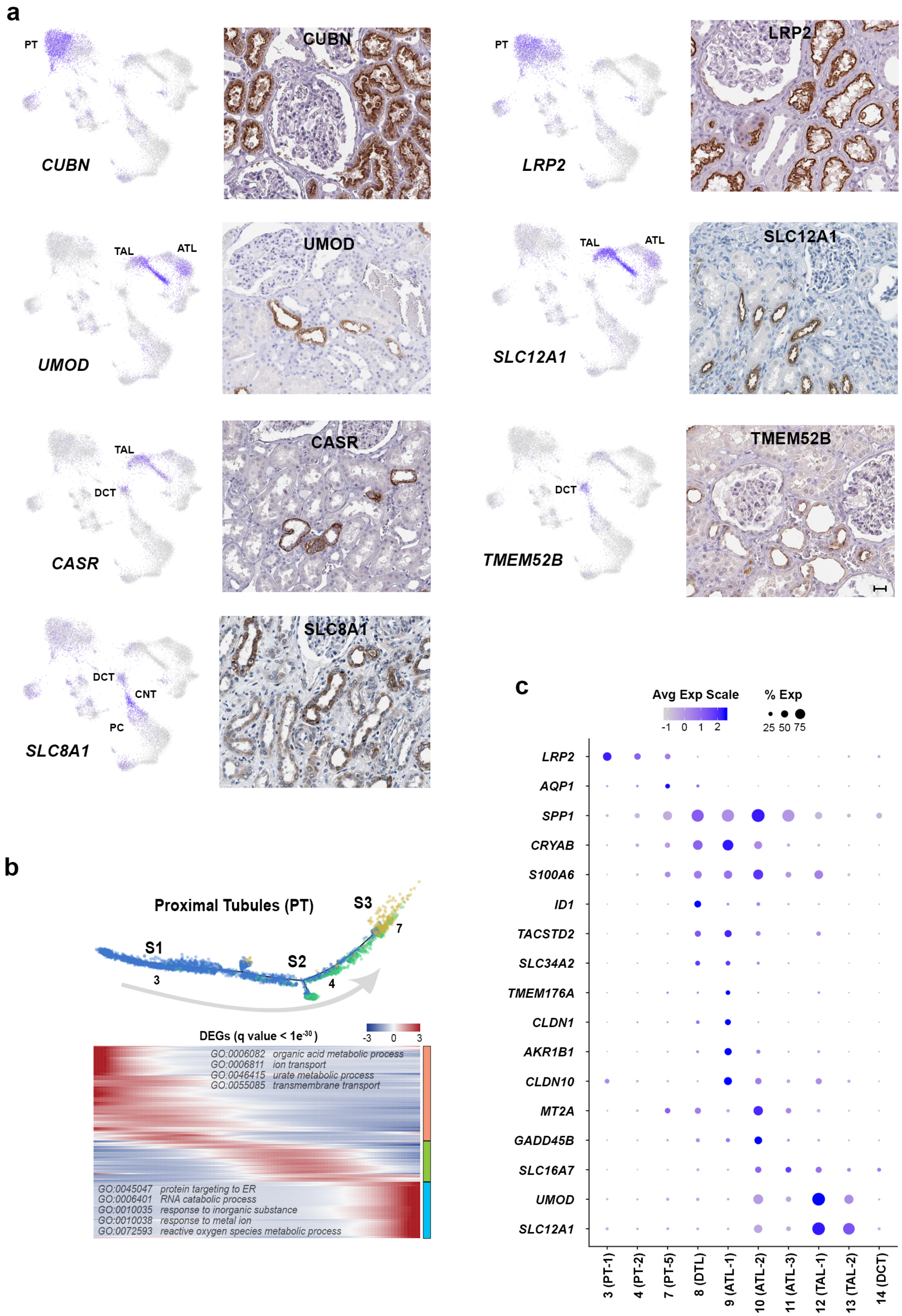

a
PT
CUBN
CUBN
PT
LRP2
LRP2
TAL
ATL
UMOD
UMOD
TAL
ATL
SLC12A1
SLC12A1
TAL
DCT
CASR
CASR
DCT
TMEM52B
TMEM52B
DCT
CNT
PC
SLC8A1
SLC8A1
b
Proximal Tubules (PT)
S1
S2
S3
3
4
7
DEGs (q value < 1e-30)
-3 0 3
GO:0006082 organic acid metabolic process
GO:0006811 ion transport
GO:0046415 urate metabolic process
GO:0055085 transmembrane transport
GO:0045047 protein targeting to ER
GO:0006401 RNA catabolic process
GO:0010035 response to inorganic substance
GO:0010038 response to metal ion
GO:0072593 reactive oxygen species metabolic process
c
Avg Exp Scale
-1 0 1 2
% Exp
25 50 75
LRP2
AQP1
SPP1
CRYAB
S100A6
ID1
TACSTD2
SLC34A2
TMEM176A
CLDN1
AKR1B1
CLDN10
MT2A
GADD45B
SLC16A7
UMOD
SLC12A1
3 (PT-1)
4 (PT-2)
7 (PT-5)
8 (DTL)
9 (ATL-1)
10 (ATL-2)
11 (ATL-3)
12 (TAL-1)
13 (TAL-2)
14 (DCT)

Supplementary Figure 8. Progressive renal tubule cell types. a. UMAP plots as in **Fig. 1b** showing relative expression levels (scaled from low - gray to high - blue) of associated markers in indicated cell types and corresponding protein immunostainings (Human Protein Atlas[1], **Supplementary Data 16**). Scale bar indicates 25 µm. **b.** Trajectory analysis of main PT clusters supporting their S1, S2 and S3 PT segment identities as shown in **Fig. 3b**. Heatmap of expression (row Z-scores) for genes differentially expressed (q value < $1e^{-30}$, see Methods) along the proximal tubule trajectory shown in **(b)** that were grouped into three gene sets by hierarchical clustering (**Supplementary Data 10,** see Methods). Associated gene ontologies are indicated for gene sets 1 and 3. **c.** Dot plot of average marker gene expression values (log scale) and percentage of nuclei expressing these genes for a subset of nephron clusters (**Fig. 1b**). Genes shown are those found to be differentially expressed (**Supplementary Data 7)** and that exhibit distinct segment-specific expression in prior studies **(Supplementary Data 5).**

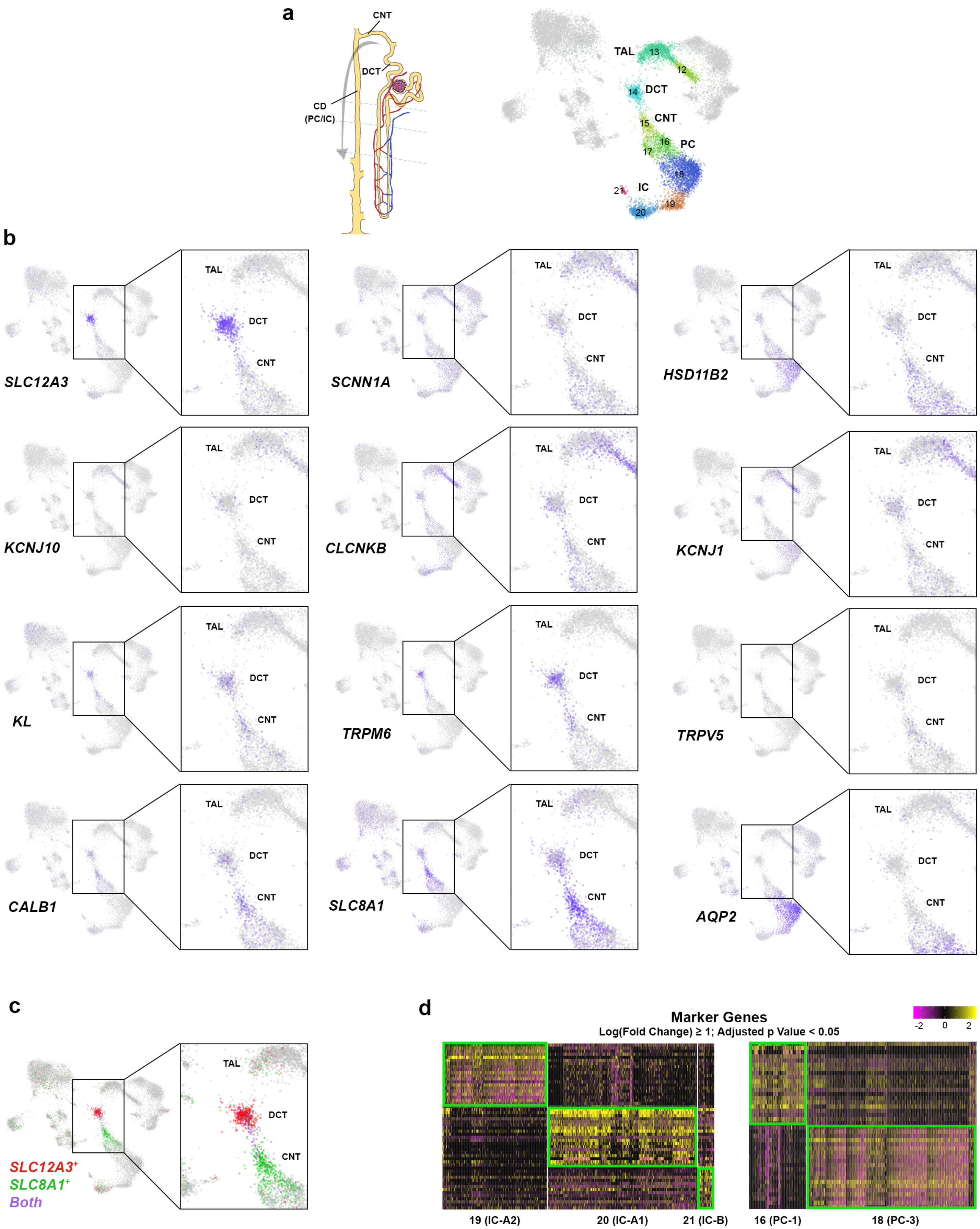

a
CNT
DCT
CD
(PC/IC)
TAL
13
12
14
DCT
15
CNT
16
17
PC
18
21
IC
20
19
b
SLC12A3
SCNN1A
HSD11B2
KCNJ10
CLCNKB
KCNJ1
KL
TRPM6
TRPV5
CALB1
SLC8A1
AQP2
TAL
DCT
CNT
c
SLC12A3+
SLC8A1+
Both
d
Marker Genes
Log(Fold Change) ≥ 1; Adjusted p Value < 0.05
-2
0
2
19 (IC-A2)
20 (IC-A1)
21 (IC-B)
16 (PC-1)
18 (PC-3)

Supplementary Figure 9. Distinct DCT, CNT and CD expression profiles. a. Schematic of the kidney nephron and UMAP as shown in **Fig. 1b** showing relevant cell populations. **b.** UMAP plots as in **Fig. 1b** showing relative expression levels (scaled from low - gray to high - blue) of genes associated with DCT and CNT function. **c.** UMAP plots as in **Fig. 1b** showing high expressing cells for the DCT marker *SLC12A3* (red) or the CNT marker *SLC8A1* (green), or both (purple). **d.** Heatmap of expression of marker genes defining IC and PC clusters using criteria indicated and for differentially expressed genes found in **Supplementary Data 11-12.**

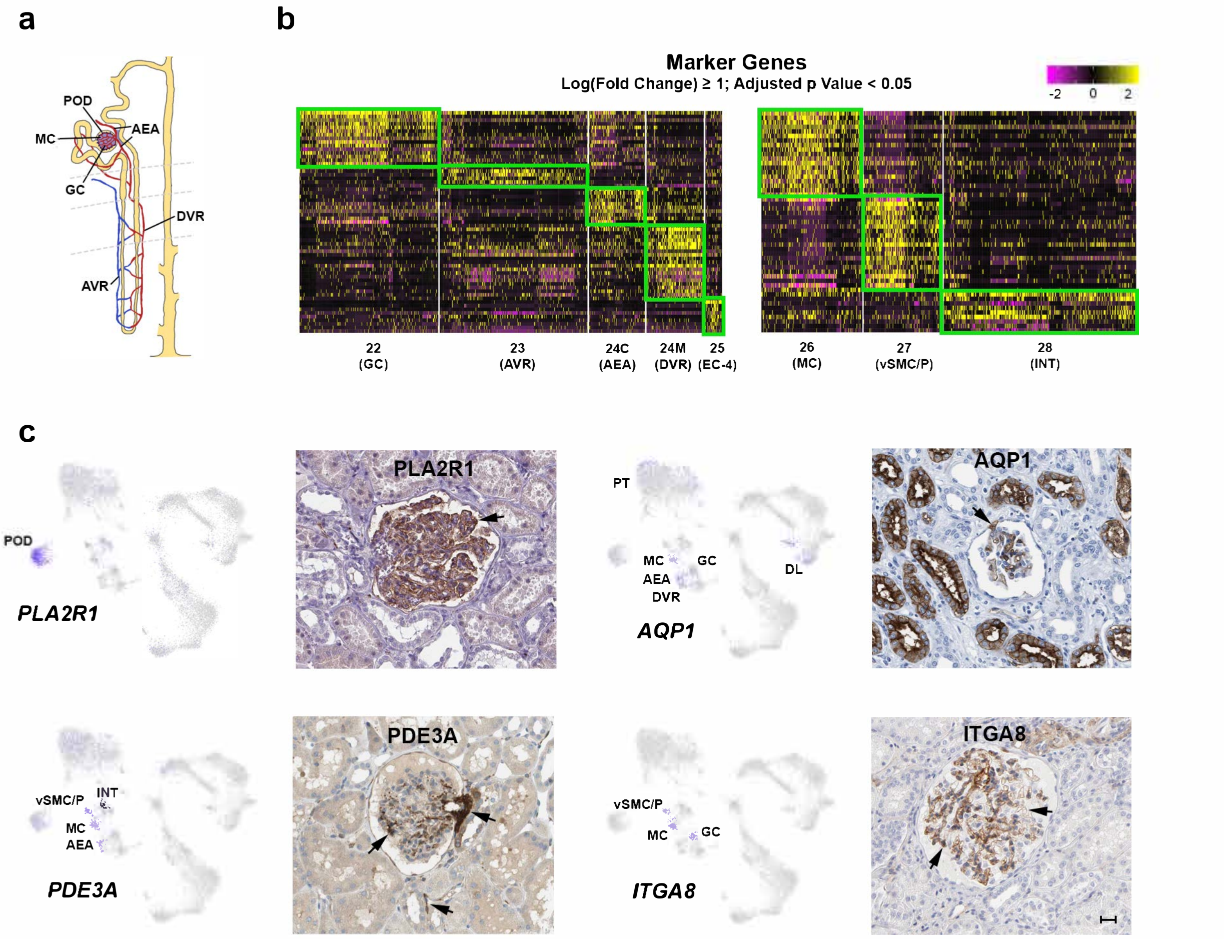

Supplementary Figure 10. Endothelial and interstitial populations. a. Schematic of the kidney nephron showing relevant cell populations. **b.** Heatmap of expression of marker genes defining EC and INT populations using criteria indicated and for differentially expressed genes found in **Supplementary Data 13-14. c.** UMAP plots as in **Fig. 1b** showing relative expression levels (scaled from low - gray to high - blue) of associated markers in indicated cell types and corresponding protein immunostainings (Human Protein Atlas[1], **Supplementary Data 16**). Arrows indicate representative protein localization. Scale bar indicates 25 μm.

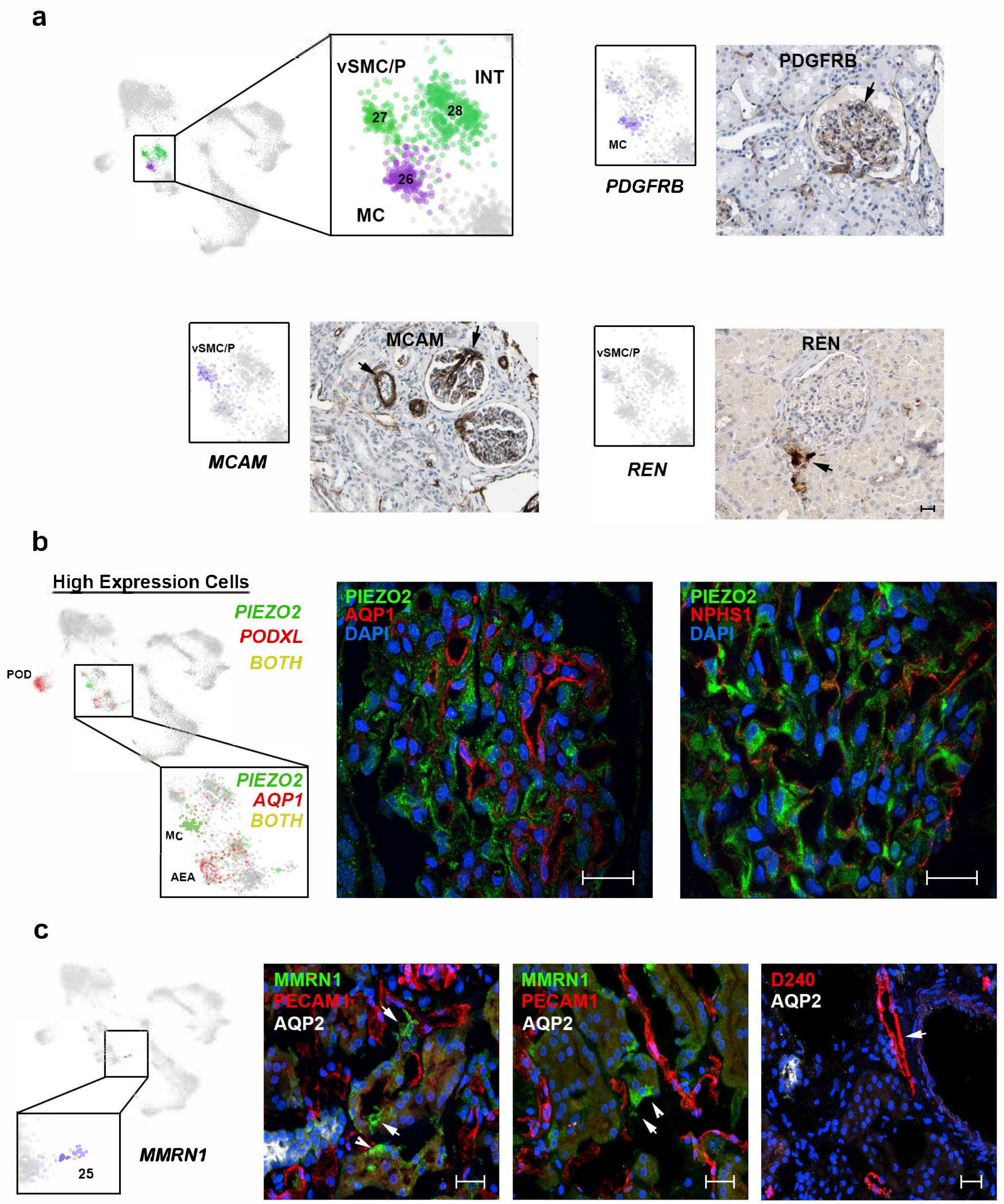

Supplementary Figure 11. Endothelial and interstitial populations. a. UMAP plots as in **Fig. 1b** showing relative expression levels (scaled from low - gray to high - blue) of associated markers in indicated cell types and corresponding protein immunostainings (Human Protein Atlas[1], **Supplementary Data 16**). Arrows indicate representative protein localization. Scale bar indicates 25 µm. **b.** UMAP plots as in **Fig. 1b** indicating high expressing cells for *PIEZO2* (green) and *PODXL* or *AQP1* (red). Cells expressing both are indicated in yellow. Protein fluorescent immunostaining (**Supplementary Data 17**) for PIEZO2 in MC, AQP1 in GCs and NPHS1 in POD. Scale bar indicates 20 µm. **d.** UMAP plots as in **Fig. 1b** showing relative expression levels (scaled from low - gray to high - blue) of *MMRN1* specific to EC-4 (cluster 25). Protein fluorescent immunostaining (**Supplementary Data 17**) for MMRN1, vascular marker PECAM1 (CD31), PC marker AQP2 and lymphatic marker D240. Closed arrows indicate MMRN1 extra-tubular staining in interstitial vessels, open arrows indicate MMRN1 intra-tubular staining. Scale bar indicates 25 µm.

Tissue Processing Pipeline

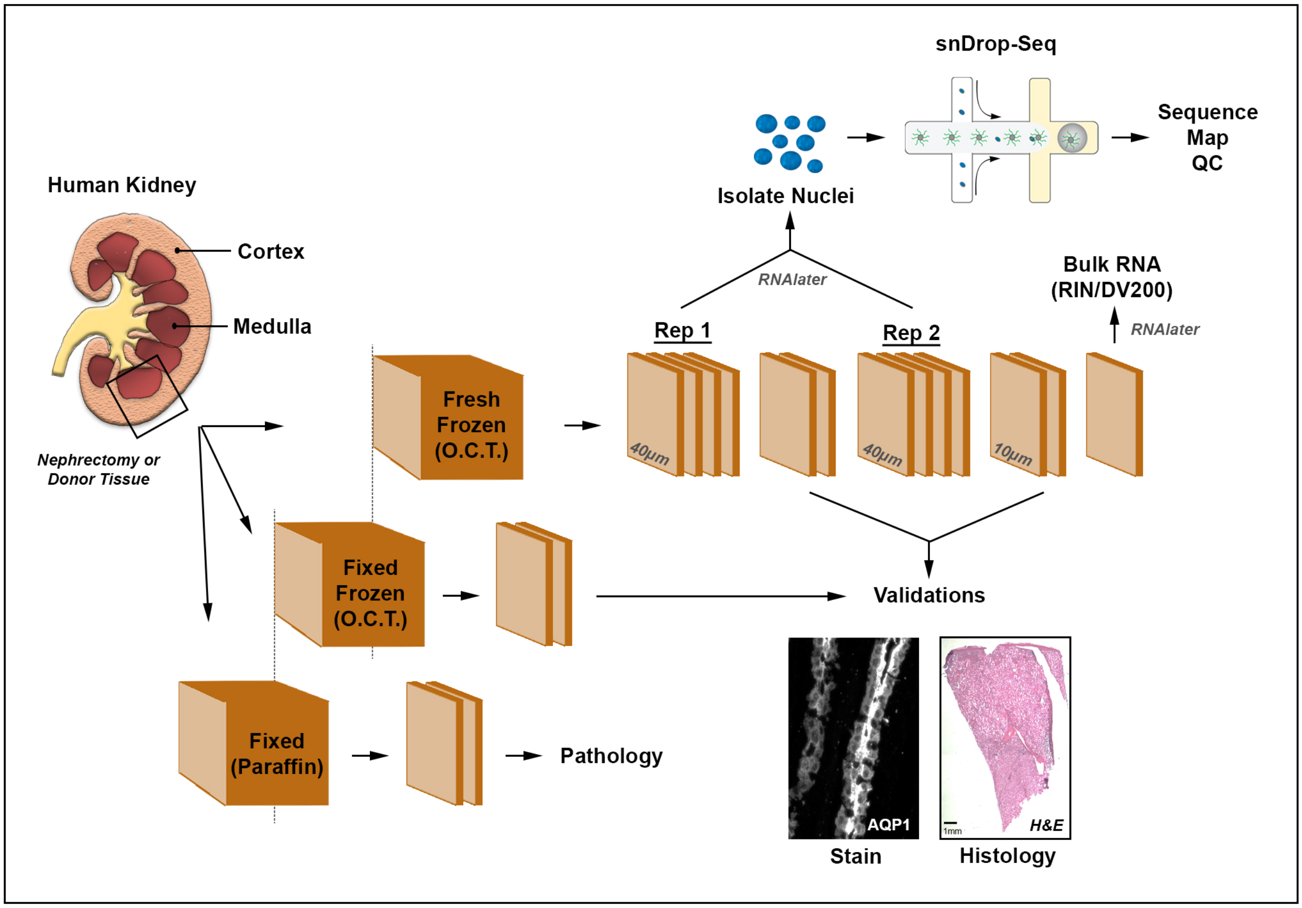

Supplementary Figure 12. Optimized tissue processing pipeline. Overview of pipeline applied to adult human kidney. Tissue is segmented into three blocks: paraffin fixed for pathological assessment; fixed and O.C.T. embedded/frozen for protein immunostaining validation/spatial registration assays; unfixed and O.C.T. embedded/frozen for single nucleus assays, bulk RNA assessment and validation/spatial registration assays. Frozen tissues permit parallel sections to be used in multiple complementary assays, specifically: 1) Thick sections are transferred to RNAlater for nuclei isolation and snDrop-Seq; 2) adjacent thin sections are used for histological assessment for tissue integrity and composition; 3) parallel thick sections can be used for technical replicates and increased sampling depth; 4) parallel thick section can be transferred to RNAlater for bulk RNA isolation and quality assessment (RNA integrity number or RIN, DV200) and for bulk RNA-seq.

Supplementary Note 1

Kidney Precision Medicine Project Consortium

Theodore Alexandov[1], Charles Alpers[2], Chris Anderton[3], Paul Appelbaum[4], Joseph Ardayfio[5], Tanima Arora[6], Tarek Ashkar[7], Mark Aulisio[8], Evren Azeloglu[9], Olivia Balderes[4], Ulysses Balis[10], Jonathan Barasch[4], Laura Barisoni[11], Daria Barwinska[7], Jack Bebiak[7], Kristina Blank[2], Andrew Bomback[4], Mary Bray[2], Keith Brown[12], Will Bush[13], Taneisha Campbell[10], Catherine Campbell[14], Leslie Cooperman[13], Dana Crawford[13], Vivette D'agati[4], Pierre Dagher[7], Ian De Boer[2], Ashveena Dighe[2], Dejan Dobi[15], Kenneth Dunn[7], Michael Eadon[7], Michele Elder[16], Michael Ferkowicz[7], Malia Fullerton[2], Yury Goltsev[17], Nir Hacohen[18], Daniel Hall[16], Habib Hamidi[10], Lynda Hayashi[12], Cijang (John) He[9], Oliver He[10], Susan Hedayati[14], Leal Herlitz[13], Jonathan Himmelfarb[2], Jeffrey Hodgin[10], Paul Hoover[18], Ravi Iyengar[9], Nichole Jefferson[2], Maria Joanes[15], John Kellum[16], Katherine Kelly[7], Asra Kermani[14], Krzysztof Kiryluk[4], Richard Knight[19], Robert Koewler[20], Matthias Kretzler[10], Mary Kruth[16], Zoltan Laszik[15], Stewart Lecker[22], Simon Lee[14], Chrysta Lienczewski[10], Christopher Lu[14], Randy Luciano[6], Laura Mariani[10], Robyn Mcclelland[2], Gearoid Mcmahon[22], Karla Mehl[4], Steven Menez[23], Raji Menon[10], Tyler Miller[14], Orson Moe[14], Dennis Moledina[6], Sean Mooney[2], Raghav Murugan[16], Garry Nolan[17], George (Holt) Oliver[24], John O'toole[13], Edgar Otto[10], Paul Palevsky[16], Chirag Parikh[23], Samir Parikh[25], Christopher Park[2], Harold Park[14], Ljiljana Pasa-Tolic[3], Emilio Poggio[13], Parmjeet Randhawa[16], Glenda Roberts[2], Sylvia Rosas[5], Avi Rosenberg[6], Matthew Rosengart[16], Kamalanathan Sambandam[14], Francisco Sanchez[14], Minnie Sarwal[15], John Saul[2], Jennifer Schaub[10], Andrew Schroeder[15], Rachel Sealfon[26], John Sedor[13], Ning (Sunny) Shang[4], Stuart Shankland[2], Kumar Sharma[27], Anna Shpigel[23], Tara Sigdel[15], Sunny Sims-Lucas[16], Rebecca Steck[10], Mary Stefanick[16], Isaac Stillman[22], Stacy Stull[16], Edith Christine Stutzke[12], Swastika Sur[15], Timothy Sutton[7], Jose Torrealba[14], Robert Toto[14], Olga Troyanskaya[26], Mitchell Tublin[16], Katherine Tuttle[12], Ugwuowo Ugochukwu[6], Miguel Vasquez[14], Dusan Velickovic[3], Pam Villalobos[23], Sus Waikar[22], Nancy Wang[14], Astrid Weins[22], Chenhua Weng[4], Mark Williams[5], Kayleen Williams[2], Francis Perry Wilson[6], Seth Winfree[7], Lorenda Wright[27], Guanshi Zhang[27], Blue B. Lake[28], Masato Hoshi[20], Diane Salamon[20], Amanda Knoten[20], Anitha Vijayan[20], Joseph Gaut[20], Kun Zhang[28], Sanjay Jain[20]

[1]European Molecular Biology Laboratory, Germany
[2]University of Washington, WA
[3]Pacific Northwest National Laboratories, WA
[4]Columbia University, NY
[5]Joslin Diabetes Center, MA
[6]Yale University, CT
[7]Indiana University, IN
[8]Case Western Reserve, OH
[9]Mount Sinai, NY
[10]University of Michigan, MI
[11]Duke University, NC
[12]Providence Health, WA
[13]Cleveland Clinic, OH
[14]UT Southwestern, TX
[15]UC San Francisco, CA
[16]University of Pittsburgh, PA
[17]Stanford University, CA
[18]Broad Institute, MA
[19]American Association of Kidney Patients, FL
[20]Washington University St. Louis, MO
[21]Beth Israel Deaconess, MA
[22]Brigham & Women's Hospital, MA
[23]John Hopkins University, MD
[24]Parkland Hospital, TX
[25]Ohio State University
[26]Princeton University, NJ
[27]UT Health San Antonio, TX
[28]UC San Diego, CA

REFERENCES

1. Uhlen, M. *et al.* Proteomics. Tissue-based map of the human proteome. *Science* **347**, 1260419 (2015).
