# Supplementary material for: A single-nucleus RNA-sequencing pipeline to decipher the molecular anatomy and pathophysiology of human kidneys
Source: Nat Commun. 2019 Jun 27;10:2832. doi: 10.1038/s41467-019-10861-2 (PMC6597610; doi:10.1038/s41467-019-10861-2)
Supplement: Supplementary file 2 — Description of Additional Supplementary Files [file 41467_2019_10861_MOESM2_ESM.docx]

**Description of Supplementary Files**

**File Name:** Supplementary Data 1

**Description:** Sample metadata.

**File Name:** Supplementary Data 2

**Description:** snDrop-seq sample information.

**File Name:** Supplementary Data 3

**Description:** snDrop-seq experiment summary.

**File Name:** Supplementary Data 4

**Description:** snDrop-seq single nuclei metadata table (Post-QC Nuclei).

**File Name:** Supplementary Data 5

**Description:** Cluster annotations.

**File Name:** Supplementary Data 6

**Description:** Cluster proportions.

**File Name:** Supplementary Data 7

**Description:** Differentially expressed genes between all clusters.

**File Name:** Supplementary Data 8

**Description:** Differentially expressed chronic kidney disease- and hypertensionassociated genes between all clusters.

**File Name:** Supplementary Data 9

**Description:** SWNE expression tables.

**File Name:** Supplementary Data 10

**Description:** Proximal tubule trajectory analysis.

**File Name:** Supplementary Data 11

**Description:** Differentially expressed genes between CD-IC clusters.

**File Name:** Supplementary Data 12

**Description:** Differentially expressed genes between CD-PC clusters.

**File Name:** Supplementary Data 13

**Description:** Differentially expressed genes between EC clusters.

**File Name:** Supplementary Data 14

**Description:** Differentially expressed genes between INT clusters.

**File Name:** Supplementary Data 15

**Description:** Primers used in snDrop-seq.

**File Name:** Supplementary Data 16

**Description:** Protein staining reference table (Human Protein Atlas).

**File Name:** Supplementary Data 17

**Description:** Protein fluorescent immunostaining reference table.
