# Supplementary material for: A single-nucleus RNA-sequencing pipeline to decipher the molecular anatomy and pathophysiology of human kidneys
Source: Nat Commun. 2019 Jun 27;10:2832. doi: 10.1038/s41467-019-10861-2 (PMC6597610; doi:10.1038/s41467-019-10861-2)
Supplement: Supplementary file 20 — Reporting Summary [file 41467_2019_10861_MOESM20_ESM.pdf]

nature research

Corresponding author(s): kzhang@bioeng.ucsd.edu; sanjayjain@wustl.edu

Last updated by author(s): May 24, 2019

Reporting Summary

Nature Research wishes to improve the reproducibility of the work that we publish. This form provides structure for consistency and transparency in reporting. For further information on Nature Research policies, see Authors & Referees and the Editorial Policy Checklist.

Statistics

For all statistical analyses, confirm that the following items are present in the figure legend, table legend, main text, or Methods section.

n/a	Confirmed	
☐	☒	The exact sample size (*n*) for each experimental group/condition, given as a discrete number and unit of measurement
☐	☒	A statement on whether measurements were taken from distinct samples or whether the same sample was measured repeatedly
☐	☒	The statistical test(s) used AND whether they are one- or two-sided<br>*Only common tests should be described solely by name; describe more complex techniques in the Methods section.*
☐	☒	A description of all covariates tested
☐	☒	A description of any assumptions or corrections, such as tests of normality and adjustment for multiple comparisons
☐	☒	A full description of the statistical parameters including central tendency (e.g. means) or other basic estimates (e.g. regression coefficient) AND variation (e.g. standard deviation) or associated estimates of uncertainty (e.g. confidence intervals)
☐	☒	For null hypothesis testing, the test statistic (e.g. *F*, *t*, *r*) with confidence intervals, effect sizes, degrees of freedom and *P* value noted<br>*Give P values as exact values whenever suitable.*
☒	☐	For Bayesian analysis, information on the choice of priors and Markov chain Monte Carlo settings
☐	☒	For hierarchical and complex designs, identification of the appropriate level for tests and full reporting of outcomes
☐	☒	Estimates of effect sizes (e.g. Cohen's *d*, Pearson's *r*), indicating how they were calculated

Our web collection on statistics for biologists contains articles on many of the points above.

Software and code

Policy information about availability of computer code

Data collection	Mapping and digital expression matrix generation of unique molecular identifier (UMI) counts for all genes and all cell barcodes, was performed largely as described (http://mccarrolllab.com/wp- content/uploads/2016/03/Drop-seqAlignmentCookbookv1.2Jan2016.pdf) with additional correction steps for processing nuclei data as outlined in the Methods and provided as supplementary software (https://github.com/chensong611/Dropseq_pipeline).
Data analysis	Data analysis as described in Methods was performed in R (version 3.4.4, https://cran.r-project.org/) using the following software packages: PAGODA2 (https://github.com/hms-dbmi/pagoda2); Seurat (version 2.3.4, https://satijalab.org/seurat/); SWNE (https://yanwu2014.github.io/swne/); and Monocle (Version 2.6.4, http://cole-trapnell-lab.github.io/monocle-release/)

For manuscripts utilizing custom algorithms or software that are central to the research but not yet described in published literature, software must be made available to editors/reviewers. We strongly encourage code deposition in a community repository (e.g. GitHub). See the Nature Research guidelines for submitting code & software for further information.

Data

Policy information about availability of data

All manuscripts must include a data availability statement. This statement should provide the following information, where applicable:

- Accession codes, unique identifiers, or web links for publicly available datasets
- A list of figures that have associated raw data
- A description of any restrictions on data availability

Raw snDrop-seq RNA sequencing data and annotated digital expression matrices are available from the NCBI Gene Expression Omnibus, accession code GSE121862 [https://www.ncbi.nlm.nih.gov/geo/query/acc.cgi?acc=GSE121862]. All relevant data are also available from the corresponding authors upon request. Previously published data that was used in this study are also available from NCBI GEO: GSE107585 [ftp://ftp.ncbi.nlm.nih.gov/geo/series/GSE107nnn/GSE107585/suppl/GSE107585_Mouse_kidney_single_cell_datamatrix.txt.gz]; GSE109564 [ftp://ftp.ncbi.nlm.nih.gov/geo/series/GSE109nnn/GSE109564/suppl/GSE109564_Kidney.biopsy.dge.txt.gz]; GSE114156 [ftp://ftp.ncbi.nlm.nih.gov/geo/series/GSE114nnn/GSE114156/suppl/GSE114156_Human.kidney.dge.txt.gz]. Source data underlying Fig. 1d, Supplementary Figures 1a, 1b, 5e, 6b are provided as Source Data File 1. Source data underlying Fig. 1b, 2, 3a-d, 3f, 4a-d, 5a-b, 5e-f,

5h, 6a and Supplementary Figures 2-7, 8b-c, 9, 10b, 11 are provided as Source Data File 2. Additional phenotyping data on participants PPID 3351, 3395, 3411, 3412, 3414, 3431, 3432, 3434, 3435, 3444 is are available upon reasonable request to sanjayjain@wustl.edu. A full data availability statement has been provided in the manuscript as well.

Field-specific reporting

Please select the one below that is the best fit for your research. If you are not sure, read the appropriate sections before making your selection.

☒ Life sciences ☐ Behavioural & social sciences ☐ Ecological, evolutionary & environmental sciences

For a reference copy of the document with all sections, see nature.com/documents/nr-reporting-summary-flat.pdf

Life sciences study design

All studies must disclose on these points even when the disclosure is negative.

Sample size

Samples from 15 individuals were selected and processed using different tissue preservation and processing methods to give ~18,000 single nuclei, a number determined as sufficient for accurate cell type discovery and gene expression profiling. As outlined in Supplementary Data 3, sampling rates for different tissue processing methods were: wholeF - 1 experiment, 1 individual; wholeR: 5 experiments, 4 individuals; dissocTC: 2 experiments, 1 individual; dissocPC: 3 experiments, 3 individuals; cryoF: 17 experiments, 13 individuals; cryoW: 3 experiments, 2 individuals; cryoR: 13 experiments and 12 individuals. At least 50 post-QC nuclei per library was needed for a snDrop-seq experiment to be considered successful and for inclusion of the data in downstream analyses. A minimum of 30 nuclei per cluster was determined as sufficient for assessment of differentially expressed genes within the cluster and cell type classification.

Data exclusions

Low quality snDrop-seq data was excluded using QC filtering metrics as described in Supplementary Figure 2a and Methods. In addition, one cluster of 120 nuclei failed to show distinct marker gene expression (no genes showing within-cluster average gene expression values > 1 log fold change over averaged expression across remaining clusters) and so was excluded as possible low-quality nuclei or multiplets.

Replication

To ensure reproducibility, snDrop-seq analyses were performed on samples obtained from several different individuals (15 total) over 27 different experiments, 2 different tissue procurement sites (UMICH, WU) and 2 different regions (cortex and medulla) as detailed in Supplementary Data 2.

Randomization

The main purpose of the study was to examine different processing steps on same sample source and optimize protocols on readily available kidney tissue. As such we priotized sample collections from tumor-free nephrectomies and discarded deceased donor kidneys having detailed preanalytical parameters with regions with minimum to no underlying chronic pathology or normal kidney function. Otherwise, samples were selected randomly for different processing steps and clustering analysis was done in an unbiased manner.

Blinding

Single-nuclei were combined for clustering analyses that were performed blind using unbiased methodologies. Metadata assessment of the clusters occurred after cluster assignment and was used for interpretation of the data.

Reporting for specific materials, systems and methods

We require information from authors about some types of materials, experimental systems and methods used in many studies. Here, indicate whether each material, system or method listed is relevant to your study. If you are not sure if a list item applies to your research, read the appropriate section before selecting a response.

Materials & experimental systems

n/a	Involved in the study	
☐	☒	Antibodies
☒	☐	Eukaryotic cell lines
☒	☐	Palaeontology
☒	☐	Animals and other organisms
☐	☒	Human research participants
☒	☐	Clinical data

Methods

n/a	Involved in the study	
☒	☐	ChIP-seq
☒	☐	Flow cytometry
☒	☐	MRI-based neuroimaging

Antibodies

Antibodies used

All antibodies used in this study are described in Supplementary Data 17. Below are details per these headers. Antibody Company Cat # Raised in Dilution Fixation Objective Microscope Figure Lot #
CFH Sigma (Atlas Antibodies) HPA049176 Rabbit 1:100 4% PFA buffered with PBS 20x, 20x (zoom 3x), 100x Nikon 80i with C1 confocal system Fig. 3f A100916
CGRP Sigma C7113 Mouse 1:200 4% PFA buffered with PBS 20x, 20x (zoom 3x), 100x Nikon 80i with C1 confocal system Fig. 4h 058M4878V
KIT Sigma (Atlas Antibodies) AMAb90901 (Clone #; CL1657) Mouse 1:200 4% PFA buffered with PBS 20x, 20x (zoom 3x), 100x Nikon 80i with C1 confocal system Fig. 4f, 4g, 02778
MMRN1 LifeSpan BioScienced, Inc. LS-C334855 Rabbit 1:50 4% PFA buffered with PBS 20x, 20x (zoom 3x), 100x Nikon 80i with

C1 confocal system Fig. S7d 129163
PIEZO2 Thermo Fisher Scientific PA5-56894 Rabbit 1:100 4% PFA buffered with PBS 20x, 20x (zoom 3x), 100x Nikon 80i with C1 confocal system Fig. 5i, S7c TH2624913A
TMEM213 Sigma (Atlas Antibodies) HPA054059 Rabbit 1:100 4% PFA buffered with PBS 20x, 20x (zoom 3x), 100x Nikon 80i with C1 confocal system Fig. 4f, 4h R72052
AQP1 (1/22) Santa Cruz sc-32737 Mouse 1:50 4% PFA buffered with PBS 20x, 20x (zoom 3x), 100x Nikon 80i with C1 confocal system Fig. 3f, 5d, S7c D0918
AQP2 (C-17) Santa Cruz sc-9882 Goat 1:200 4% PFA buffered with PBS 20x, 20x (zoom 3x), 100x Nikon 80i with C1 confocal system Fig. 3f, 4f, 4h, S7d J2414
CD31 Thermo Fisher Scientific MA3100 (Clone #; HEC7) Mouse 1:50 4% PFA buffered with PBS 20x, 20x (zoom 3x), 100x Nikon 80i with C1 confocal system Fig. 5i, S7d TG259057
D240 Abcam ab77854 (Clone #; D2-40) Mouse 1:50 4% PFA buffered with PBS 20x, 20x (zoom 3x), 100x Nikon 80i with C1 confocal system Fig. S7d GR3237654-1
Nephrin (NPHS1) R&D AF-4269 Sheep 1:50 4% PFA buffered with PBS 20x, 20x (zoom 3x), 100x Nikon 80i with C1 confocal system Fig. 5i, S7c ZMU0216081
Podocalyxin Novus NBP2-25219 (Clone #; 3D3) Mouse 1:300 4% PFA buffered with PBS 20x, 20x (zoom 3x), 100x Nikon 80i with C1 confocal system Fig. 5i 5420-1P151105
NRG3 LifeSpan BioScienced, Inc. LS-B107 Rabbit 1:50 4% PFA buffered with PBS 20x, 20x (zoom 3x), 100x Nikon 80i with C1 confocal system Fig. 5d 25554

Validation

All antibodies are commercially available with validations described on the associated company websites as listed in Supplementary Data 17. Cell type-specific staining is supported by single-nucleus RNA-seq expression profiles reported in the manuscript.

Human research participants

Policy information about studies involving human research participants

Population characteristics

This study is not an interventional or clinical trial. The relevant information for study approval are stated in the methods, also see ethics oversight below. Supplemental Data 1 lists the relevant preanalytical parameters and metadata for the patients from which the data were generated. There was no genotype information available from the participants.

Recruitment

This study was designed to generate a method for tissue processing for single nucleus sequencing of disease free kidney. Available human kidney tissues, e.g. tumor nephrectomies or discarded deceased donor kidneys, were used for this study. The metadata for these samples are located in Supplementary Data 1 and we highlight in the text that the interpretation of the data must be in consideration of these parameters.

Ethics oversight

All human samples and data were collected by the Kidney Translational Research Center (KTRC) for this study under a protocol approved by the Washington University Institutional Review Board. Informed consent was obtained for the use of data and samples for all participants at Washington University and include living patients undergoing partial or total nephrectomy or from discarded deceased kidney donors (Supplementary Data 1). Samples from University of Michigan were obtained from tumor nephrectomies harvested from consented patients by the Tissue Procurement Service as a part of the Kidney Precision Medicine Project (KPMP) consortium (https://kpmp.org/) and were approved as exempted by the University of Michigan Institutional Review Board because they were anonymized. We have complied with all relevant ethical regulations related to this study.

Note that full information on the approval of the study protocol must also be provided in the manuscript.
